# Supplementary material for: Facile fabrication of recyclable robust noncovalent porous crystals from low-symmetry helicene derivative
Source: Nat Commun. 2024 Jun 27;15:5469. doi: 10.1038/s41467-024-49865-y (PMC11211482; doi:10.1038/s41467-024-49865-y)
Supplement: Supplementary file 1 — Supplementary Information [file 41467_2024_49865_MOESM1_ESM.pdf]

Supplementary Information

Facile Fabrication of Recyclable Robust Noncovalent Porous Crystals from Low-Symmetry Helicene Derivative

Guoli Zhang[1], Jian Zhang[1], Yu Tao[2], Fuwei Gan[1], Geyu Lin[1], Juncong Liang[1], Chengshuo Shen[3,*], Yuebiao Zhang[2], Huibin Qiu[1,*]

[1]School of Chemistry and Chemical Engineering, Zhangjiang Institute for Advanced Study, Frontiers Science Center for Transformative Molecules, State Key Laboratory of Metal Matrix Composites, Shanghai Jiao Tong University, Shanghai 200240, China.
[2]Shanghai Key Laboratory of High Resolution Electron Microscopy, School of Physical Science and Technology, ShanghaiTech University, Shanghai 201210, China.
[3]School of Chemistry and Chemical Engineering, Key Laboratory of Surface & Interface Science of Polymer Materials of Zhejiang Province, Zhejiang Sci-Tech University, 310018, Hangzhou, China.

Table of Contents

1. Supplementary Methods S2

2. Characterization of Single Crystals and Powder of D6H S4

3. Adsorption Experiments S17

4. Theoretical Calculations S35

Supplementary References S38

1. Supplementary Methods

1.1 General Information

All the synthetic experiments were performed using standard Schlenk techniques unless otherwise stated. Starting materials and reagents were of AR grade quality and were purchased from commercial sources and used without further purification unless otherwise noted.

NMR spectra were recorded on a Bruker Avance III HD 500 Spectrometer. Chemical shifts were determined using residual signals of the deuterated solvents and were reported in parts per million (ppm). High-resolution mass spectrometry (HR-MS) data were recorded on a Bruker time of flight mass spectrometer (autoflex speed TOF/TOF) using matrix-assisted laser desorption/ionization (MALDI) mode. Single crystal data were recorded on a Bruker D8 Venture single crystal X-ray diffractometer with graphite-monochromatic Cu Kα (λ = 1.54178 Å) radiation. Powder X-ray diffraction (PXRD) patterns were collected by a Bruker D8 Advance X-ray diffractometer using Cu Kα (λ = 1.54178 Å) radiation. Variable-temperature powder X-ray diffraction (PXRD) was collected by a Bruker D8 Advance X-ray diffractometer using Co Kα (λ = 1.79021 Å) radiation. Differential scanning calorimetry (DSC) were performed with a DSC 2500 analyzer under nitrogen atmosphere with a temperature changing rate of 10 °C /min. Thermogravimetric analyses (TGA) were performed with an SDT-Q600 analyzer under nitrogen atmosphere with a heating rate of 10 °C /min. Vapor-phase adsorption isotherms were measured with a Belsorp-MAXIIG surface area and porosity analyzer (MicrotracBELe) and a Belsorp-Aqua3 moisture sorption analyzer (MicrotracBELe). Optical microscope photos were taken by a LEICA DM 4000 optical microscope with a CCD camera. Scanning electron microscopy (SEM) micrographs were obtained on a TESCAN MAIA3 microscope operated at 5.0 kV. The adsorption isotherms were collected by a BELSORP-MAX volumetric analyzer.

1.2 Synthesis of D6H

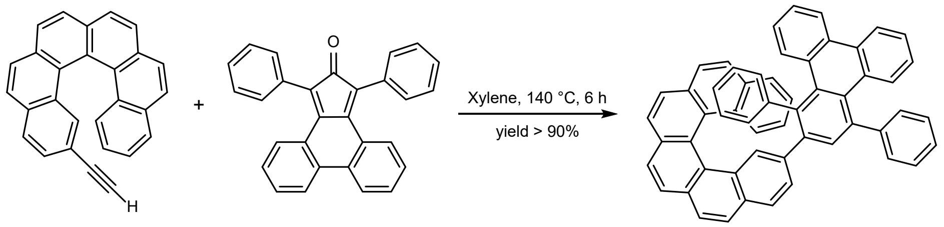

D6H was synthesized according to the literature[1]. Typically, 0.176 g of 2-ethynyl[6]helicene (0.5 mmol) and 0.191 g of phencyclone (0.5 mmol) were dissolved in 10 mL of xylene under argon. The mixture was then stirred at 140 °C for 6 hours. During the reaction, the solution color turned from blackish green to yellow with heavy white precipitate. Subsequently, the suspension was cooled to room temperature and the solvent was removed at reduced pressure. The residue was washed with ethanol and ether, and then dried to afford **D6H** as white solid (0.35 g, 95%). ^{1}H NMR (500 MHz, CS_2, 298 K): δ 8.39 (d, J = 8.4 Hz, 1H), 8.35 (d, J = 8.4 Hz, 1H), 8.09 (s, 2H), 8.07 (d, J = 8.0 Hz, 1H), 8.04 (d, J = 8.0 Hz, 1H), 8.01 (d, J = 8.4 Hz, 1H), 7.96 (d, J = 8.4 Hz, 1H), 7.92 (s, 1H), 7.90 - 7.85 (m, 2H), 7.81 (d, J = 8.4 Hz, 1H), 7.80 - 7.73 (m, 3H), 7.71 - 7.65 (m, 3H), 7.49 - 7.33 (m, 6H), 7.28 (d, J = 7.8 Hz, 1H), 7.20 - 7.04 (m, 4H), 6.95 - 6.8- (m, 4H), 6.54 (broad, 1H), 6.49 (dd, J = 7.8 Hz, J = 1.6 Hz, 1H), 6.24 (s, 1H). $^{13}C\{1H\}$ NMR spectrum could not be recorded due to the fast epimerization between different conformers. MALDI-HR-MS for $C_{56}H_{34}^{\cdot+}$ ($M^{\cdot+}$): m/z = 706.2655 (calculated), 706.2423 (found).

P-**D6H**: Starting with 35.2 mg of *P*-2-ethynyl[6]helicenes (0.1 mmol), 52.2 mg of *P*-**D6H** (yield: 74%) was obtained.

M-**D6H**: Starting with 35.2 mg of *M*-2-ethynyl[6]helicenes (0.1 mmol), 48.6 mg of *M*-**D6H** (yield: 68%) was obtained.

2. Characterization of Single Crystals and Powder of D6H

Crystallization of enantiopure D6H by solvent diffusion. Single crystals of enantiopure (*P* and *M*) **D6H** were grown in a carbon disulfide (CS_2) solution with the diffusion of a vapor of *n*-pentane. 3.0 mg of *P*-**D6H** and *M*-**D6H** were respectively dissolved in 1 mL of CS_2, and then crystalized in a vapor of *n*-pentane for 3 days. The crystals were washed with *n*-pentane and then dried.

Crystallization of D6H after melting. 10.0 mg of **D6H** was encapsulated in a glass tube with nitrogen atmosphere. The tube was heated at 300 °C for 6 hours, then cooled in a muffle furnace to 250 °C with a slow rate of 0.1 °C/min.

Supplementary Table 1 | Crystallographic data and structure refinement for the crystal of racemic **D6H** obtained by solvent diffusion (CCDC 2224852).

Empirical formula	$C_{56}H_{34}$
Formula weight	706.83
Temperature (K)	172.99
Crystal system	Trigonal
Space group	*R*-3
a (Å)	49.9332(6)
b (Å)	49.9332(6)
c (Å)	10.5479(2)
α (°)	90
β (°)	90
γ (°)	120
Volume (Å^3)	22775.9(7)
Z	18
$\rho_{calc.}$ (g·cm^{-3})	0.928
μ (mm^{-1})	0.400
F(000)	6660.0
Crystal size (mm^3)	0.1 × 0.03 × 0.02
Radiation	Cu Kα (λ = 1.54178)
2*Θ* range for data collection (°)	6.132 to 127.784
Index ranges	$-58 \leq h \leq 58$, $-58 \leq k \leq 58$, $-12 \leq l \leq 12$
Reflections collected	72124
Independent reflection	8379 [R_{int} = 0.0599, R_σ = 0.0245]
Data/restraints/parameters	8379/1/505
Goodness-of-fit on F^2	1.031
Final *R* indexes [$I \geq 2\sigma(I)$]	R_1 = 0.0358, wR_2 = 0.0888
Final *R* indexes [all data]	R_1 = 0.0454, wR_2 = 0.0940
Largest diff. peak/hole (e·Å^{-3})	0.14/−0.19

Note: CH_2Cl_2 and *n*-pentane molecules possibly exist in a disordered form, which were removed by solvent mask using Olex2.

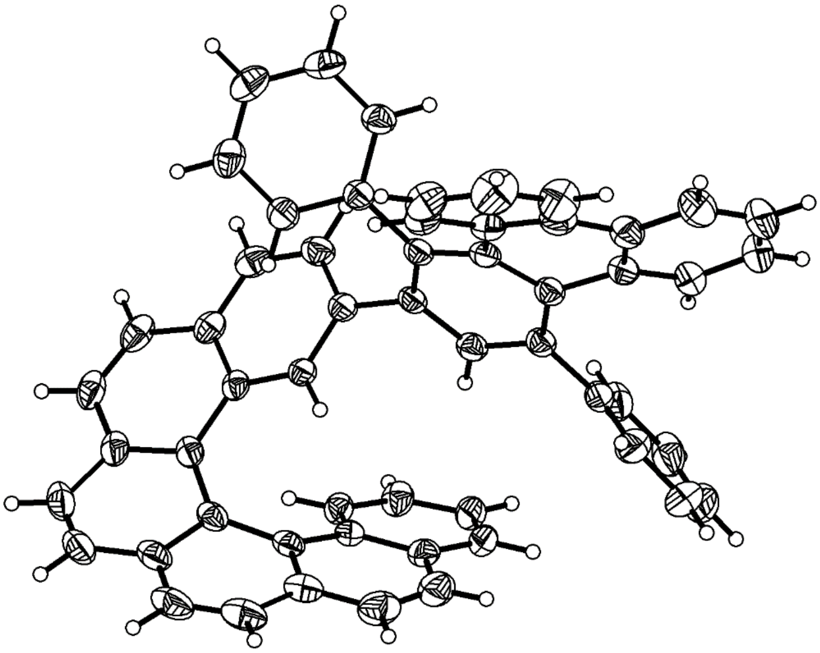

Supplementary Fig. 1 | X-ray crystallographic structure of racemic **D6H** (CCDC 2224852) (only *M* enantiomer is shown). The single crystal was obtained by solvent diffusion. Thermal ellipsoids are shown at a 50% probability level.

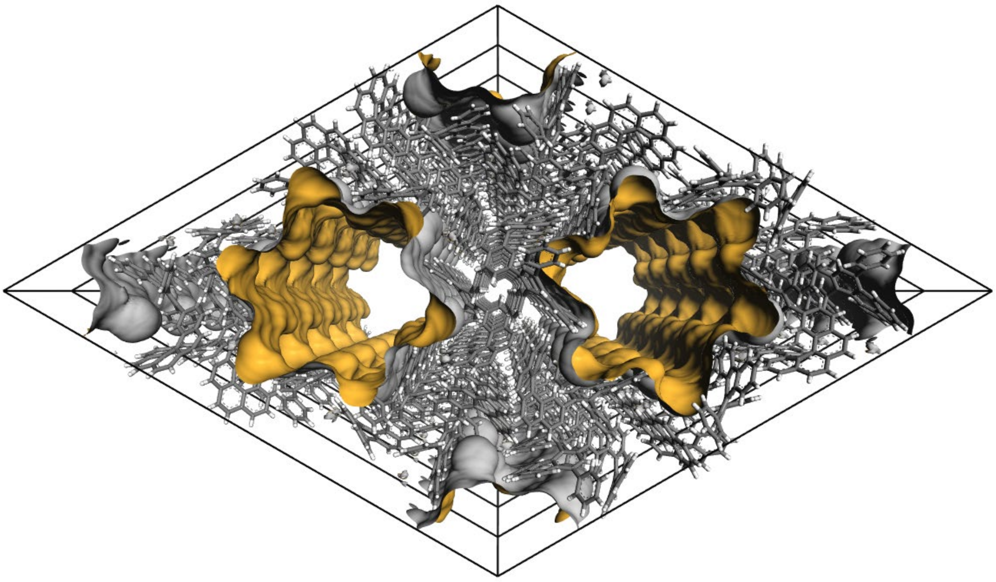

Supplementary Fig. 2 | Solvent-accessible void space generated with a probe of 1.84 Å for a crystal of racemic **D6H** obtained by solvent diffusion.

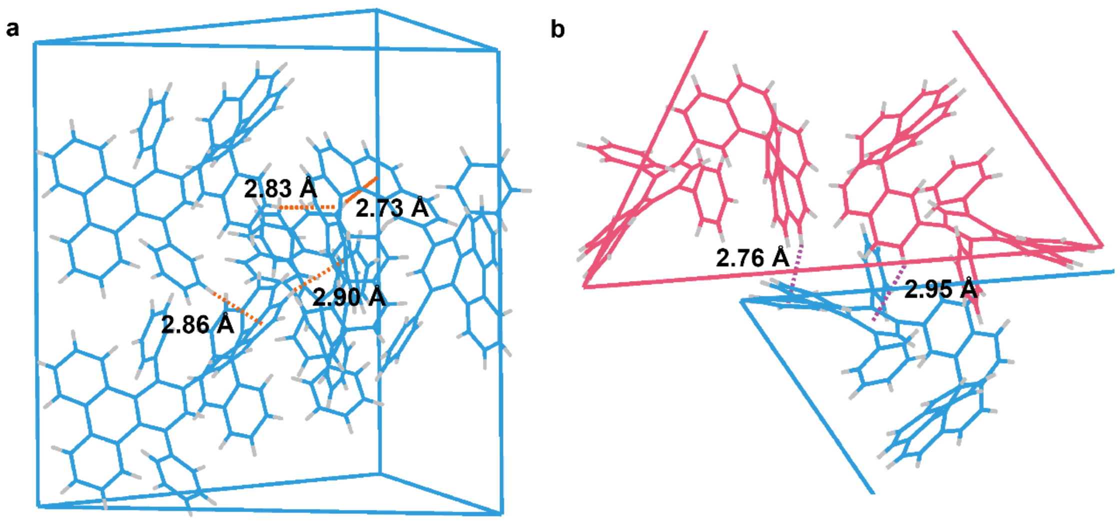

Supplementary Fig. 3 | Intermolecular interactions in a crystal of racemic **D6H** obtained by solvent diffusion. **a**, Intermolecular interactions between homochiral molecules. **b**, Intermolecular interactions between heterochiral molecules. Carbon atoms of *M*- and *P*-**D6H** are marked in blue and red, respectively.

Supplementary Table 2 | Crystallographic data and structure refinement for the crystal of *M*-**D6H** (CCDC 2268959).

Empirical formula	$C_{56.5}H_{34}S$
Formula weight	744.965
Temperature (K)	233.0
Crystal system	Triclinic
Space group	*P*1
a (Å)	10.9914(9)
b (Å)	13.2868(11)
c (Å)	14.8616(13)
α (°)	66.908(4)
β (°)	80.808(5)
γ (°)	89.602(5)
Volume (Å^3)	1967.1(3)
Z	2
$\rho_{calc.}$ (g·cm^{-3})	1.258
μ (mm^{-1})	1.024
F(000)	780.9
Crystal size (mm^3)	0.2 × 0.2 × 0.05
Radiation	Cu K$_\alpha$ (λ = 1.54178)
2Θ range for data collection (°)	6.56 to 138.52
Index ranges	$-12 \le h \le 13$, $-16 \le k \le 16$, $-17 \le l \le 17$
Reflections collected	20620
Independent reflection	12602 [R_{int} = 0.1343, R_σ = 0.1811]
Data/restraints/parameters	12602/15/1037
Goodness-of-fit on F^2	1.119
Final *R* indexes [$I \ge 2\sigma(I)$]	R_1 = 0.1089, wR_2 = 0.2850
Final *R* indexes [all data]	R_1 = 0.1773, wR_2 = 0.3409
Largest diff. peak/hole (e·Å^{-3})	1.19/−0.69

- Explanation for the Alert B

  PLAT340_ALERT_3_B Low Bond Precision on C−C Bonds 0.01208 Ang.

—— The Alert_3_B was majorly caused by the low quality of the single crystal. However, this

would not affect the structural determination in this study.

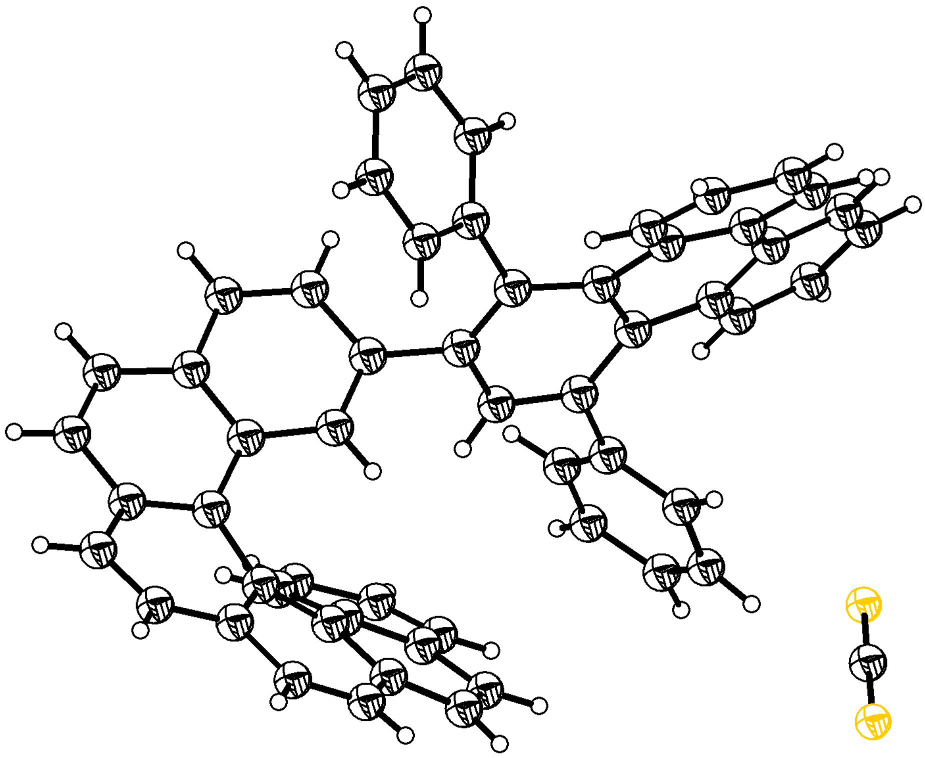

Supplementary Fig. 4 | X-ray crystallographic structure of *M*-**D6H** (CCDC 2268959). The single crystal was obtained by solvent diffusion. Thermal ellipsoids are shown at a 50% probability level.

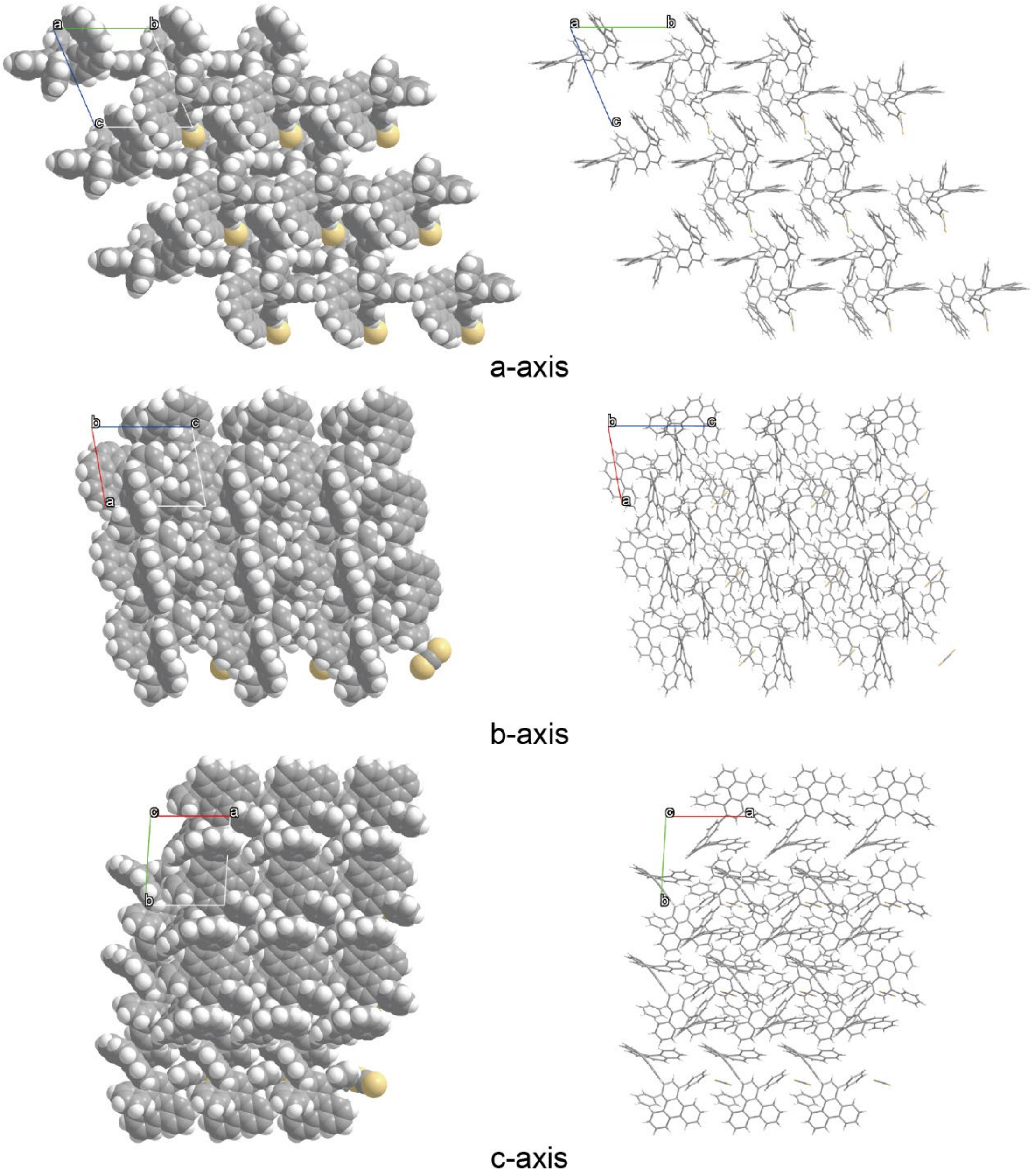

Supplementary Fig. 5 | Molecular packing of *M*-**D6H** in the crystal.

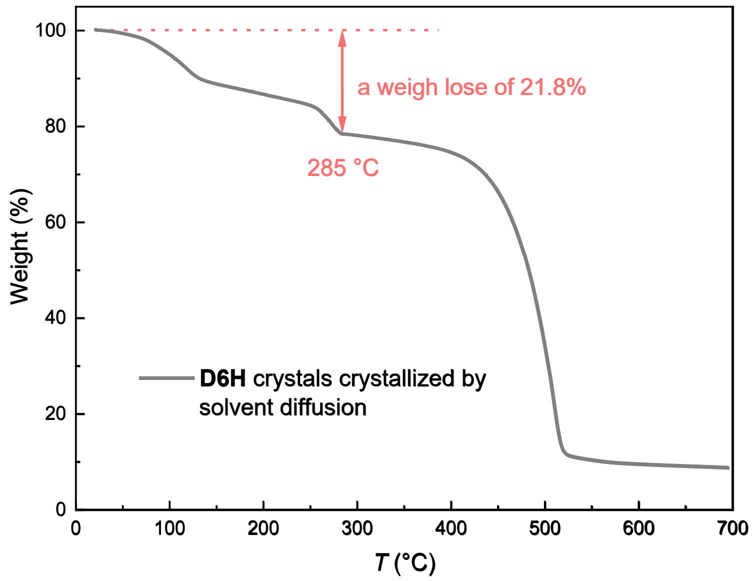

Supplementary Fig. 6 | TGA curve of the crystals of racemic **D6H** obtained by solvent diffusion.

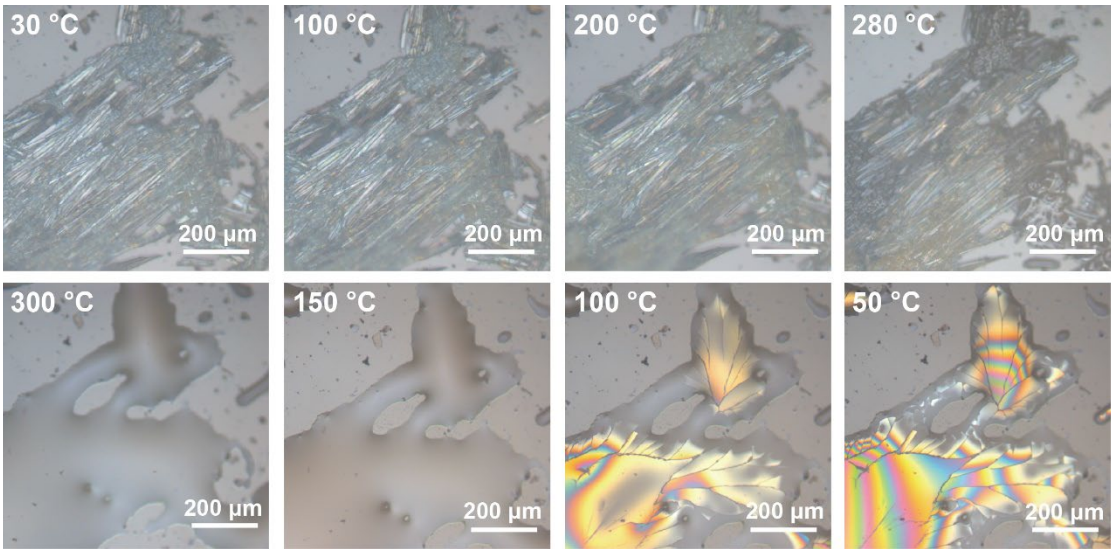

Supplementary Fig. 7 | Optical microscope images of the crystals of racemic **D6H** obtained by solvent diffusion upon heating from 30 °C to 300 °C, then cooling to 50 °C in the air.

Supplementary Table 3 | Crystallographic data and structure refinement for the crystal of racemic **D6H** obtained after melting (CCDC 2224863).

Empirical formula	$C_{56}H_{34}$
Formula weight	706.83
Temperature (K)	173.0
Crystal system	Monoclinic
Space group	$P2_1/n$
a (Å)	10.7749(2)
b (Å)	12.9241(2)
c (Å)	27.1691(5)
α (°)	90
β (°)	97.3170(10)
γ (°)	90
Volume (Å^3)	3752.65(11)
Z	4
$\rho_{calc.}$ (g·cm^{-3})	1.251
μ (mm^{-1})	0.539
F(000)	1480.0
Crystal size (mm^3)	0.1 × 0.1 × 0.1
Radiation	Cu K_α (λ = 1.54178)
2Θ range for data collection (°)	6.56 to 136.69
Index ranges	$-12 \leq h \leq 11$, $-15 \leq k \leq 15$, $-29 \leq l \leq 32$
Reflections collected	39092
Independent reflection	6848 [R_{int} = 0.0495, R_σ = 0.0296]
Data/restraints/parameters	6848/0/505
Goodness-of-fit on F^2	1.020
Final R indexes [$I \geq 2\sigma(I)$]	R_1 = 0.0351, wR_2 = 0.0834
Final R indexes [all data]	R_1 = 0.0459, wR_2 = 0.0889
Largest diff. peak/hole (e·Å^{-3})	0.15/−0.17

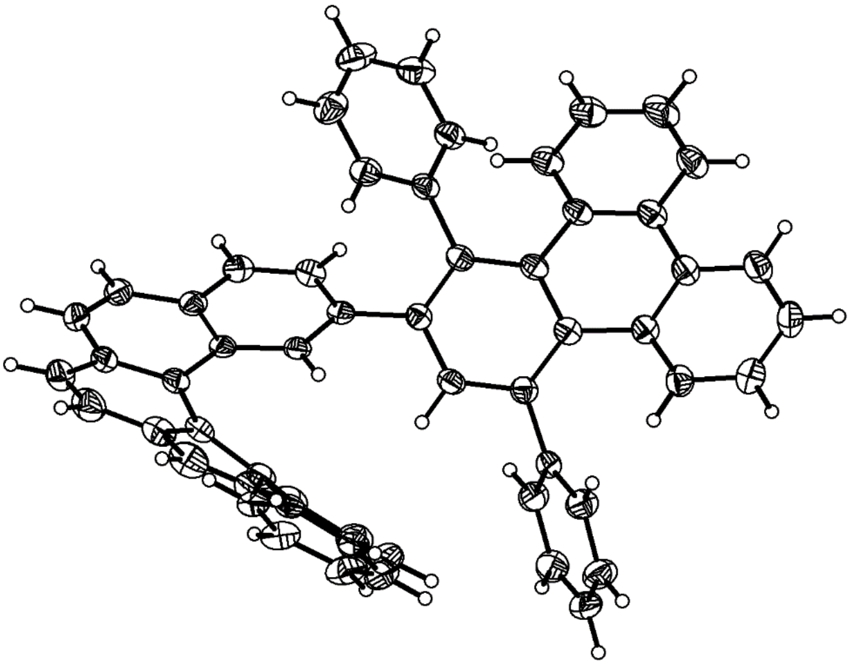

Supplementary Fig. 8 | X-ray crystallographic structure of racemic **D6H** (CCDC 2224863) (only *M* enantiomer is shown). The crystal was obtained by melting and cooling. Thermal ellipsoids are shown at a 50% probability level.

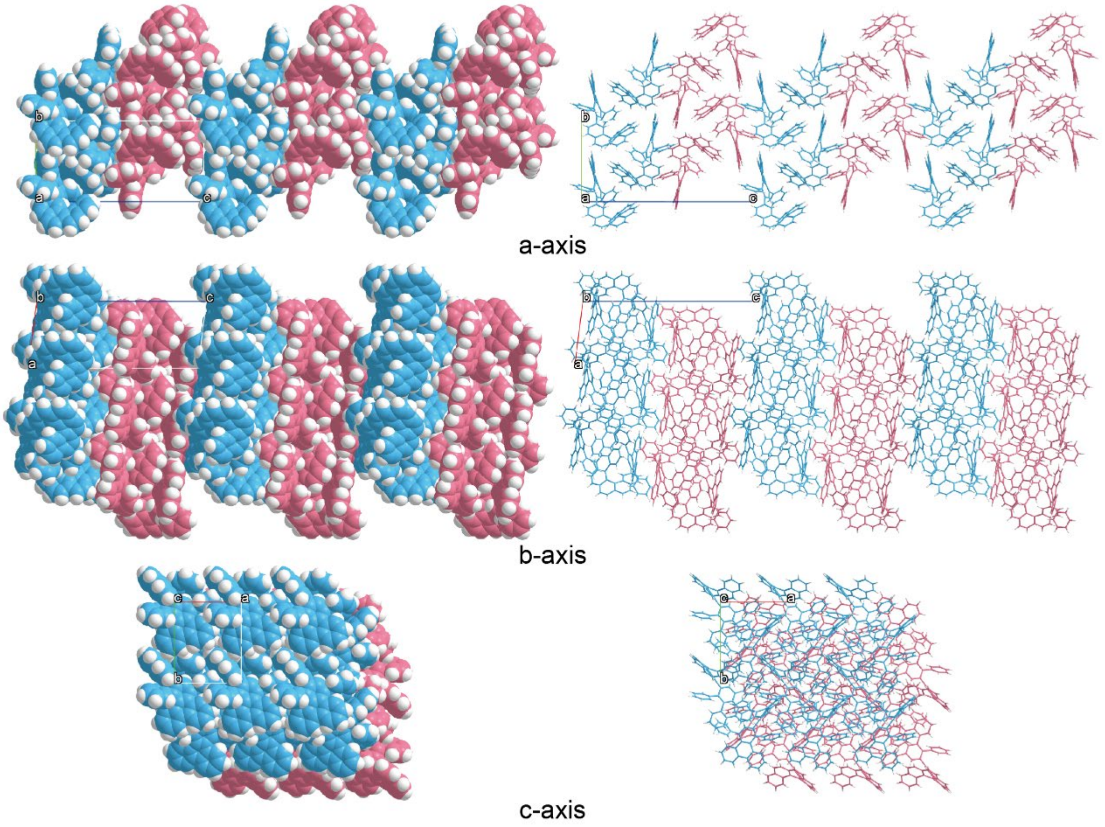

Supplementary Fig. 9 | Molecular packing of racemic **D6H** in the crystal obtained after melting.

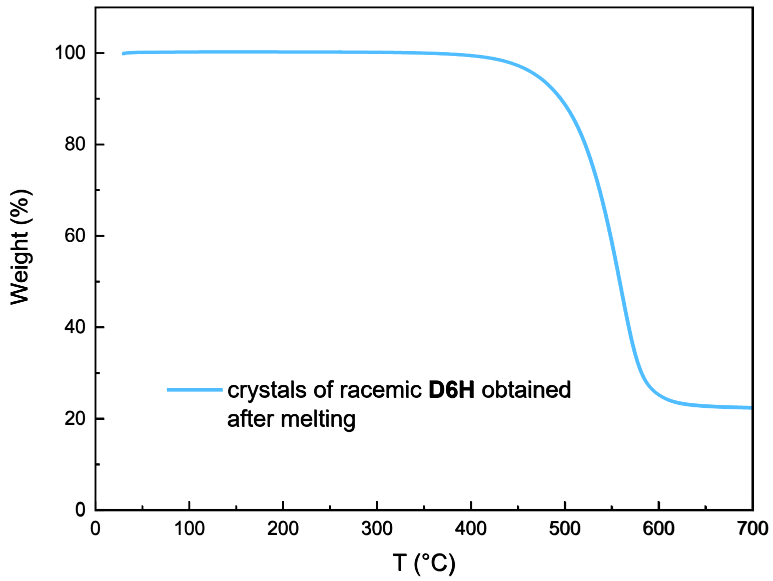

Supplementary Fig. 10 | TGA curve of the crystals of racemic **D6H** obtained after melting.

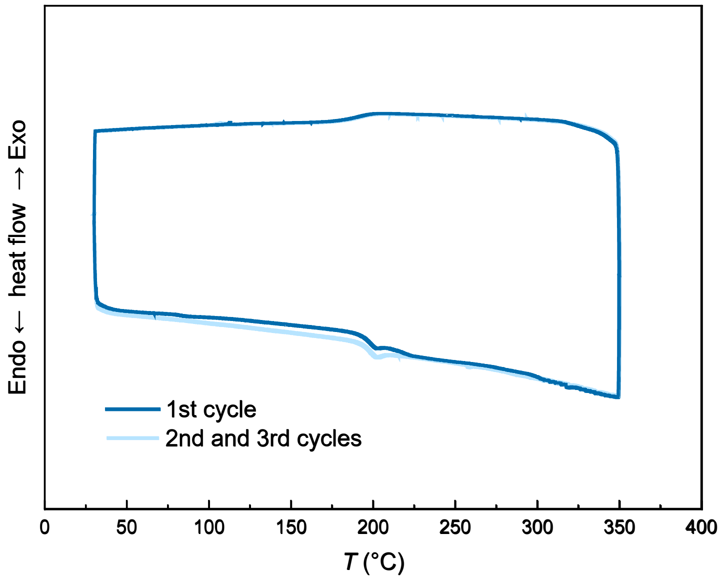

Supplementary Fig. 11 | DSC curves of the crystals of racemic **D6H** obtained after melting.

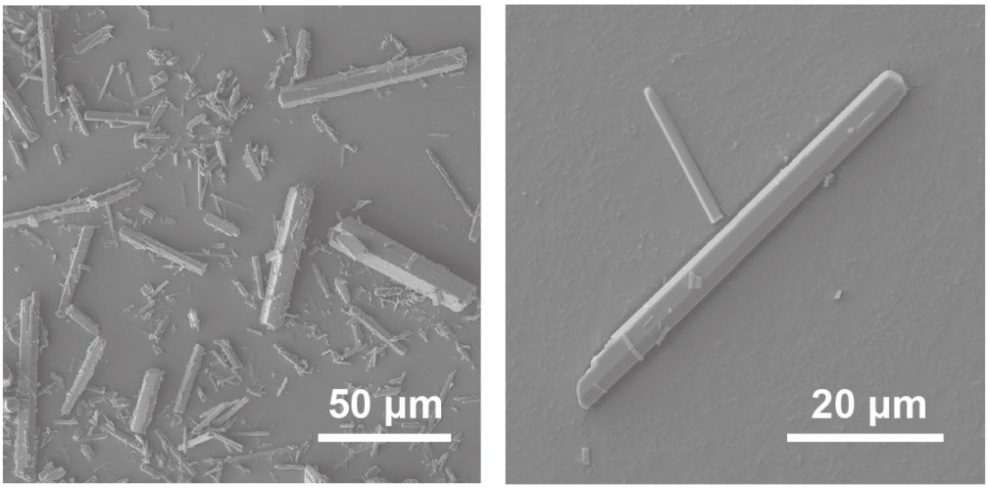

Supplementary Fig. 12 | SEM images of the crystals of racemic **D6H** obtained by solvent diffusion.

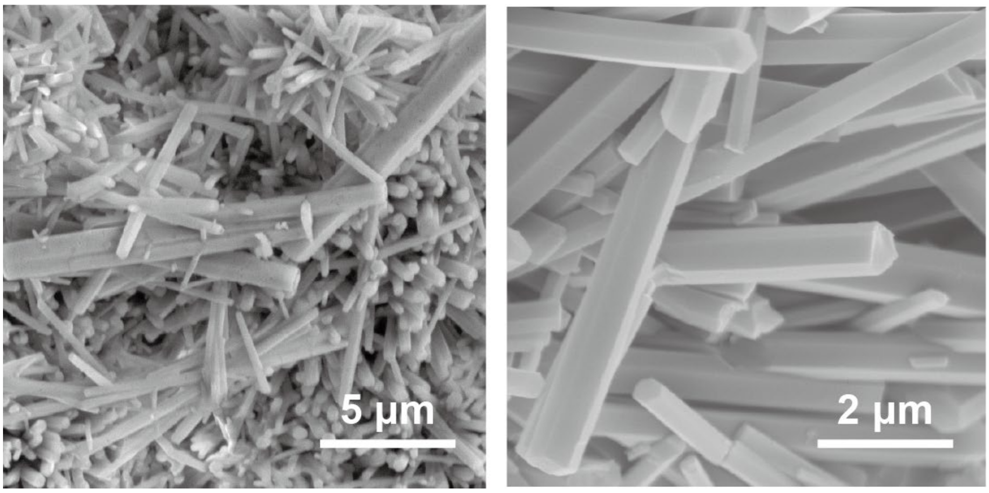

Supplementary Fig. 13 | SEM images of powder of racemic **D6H** dried from DCM.

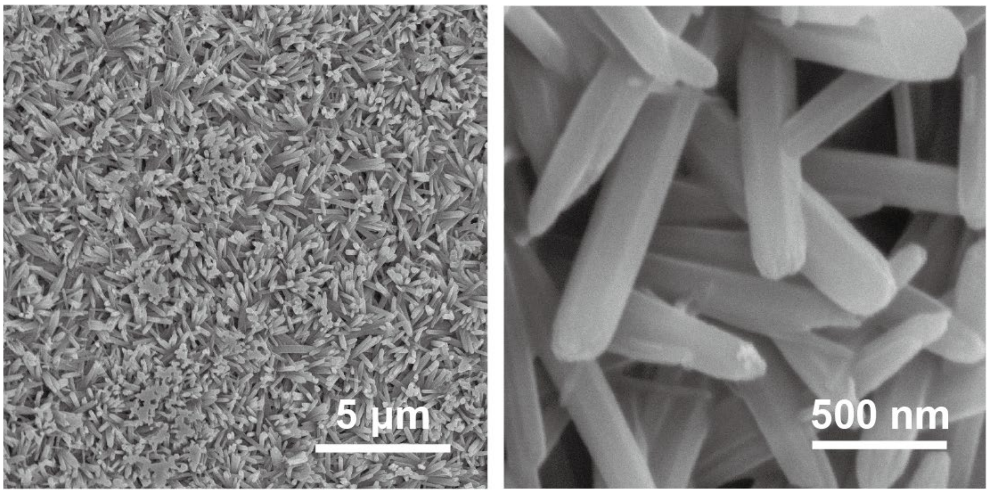

Supplementary Fig. 14 | SEM images of powder of racemic **D6H** dried from $CHCl_3$.

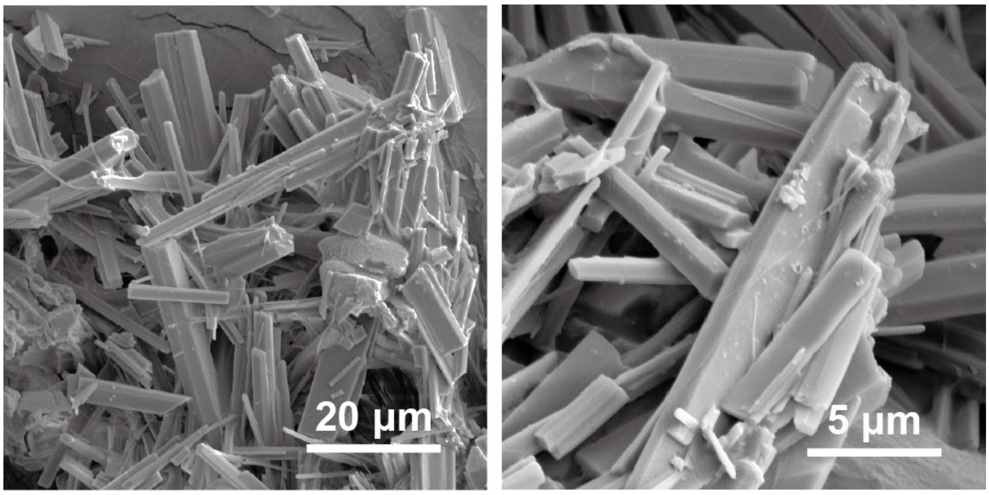

Supplementary Fig. 15 | SEM images of powder of racemic **D6H** dried from toluene.

3. Adsorption Experiments

Adsorption of various solvents. The powder of racemic **D6H** dried from DCM was activated by heating the powder at 150 °C under vacuum for 24 h. Subsequently, 10 mg of activated powder of racemic **D6H** was added into a small glass tube and then the tube was placed in a glass bottle containing 30 mL of target solvent, including THF, diisopropyl ether (DIPE), methyl tert-butyl ether (MTBE), dioxane (DOX), CCH_3, carbon tetrachloride (CCl_4), 1,2-dichloroethane ($C_2H_4Cl_2$), toluene, methanol (MeOH) and cyclohexane (CYH). The whole bottle was heated around the boiling point of the corresponding solvent for 2 days. The samples were eventually exposed in the air at room temperature overnight and the absorption capacity was finally estimated by NMR and TGA.

Adsorption of aromatic molecules. 10 mg of activated powder of racemic **D6H** was soaked in 2 mL of MeOH solutions of biphenyl (BP), azobenzene (AB) and diphenyl disulfide (DPDS) at a concentration of 25 mg/mL respectively for 2 days. Subsequently, the solid samples of **D6H** with aromatic contents were obtained by filtration and washed with MeOH for several times. The samples were dried at 50 °C overnight and the absorption capacity was finally estimated by NMR.

Cyclic adsorption-desorption experiments. Typically, 40 mg of activated powder of racemic **D6H** was added into a small glass tube and then the tube was placed in a glass bottle containing 30 mL of THF. The whole bottle was heated at 60 °C for 24 h, and 1 mg of sample was taken out for NMR test. The rest sample was desorbed by heating at 150 °C under vacuum for 24 h, and then placed back in the glass bottle for subsequent adsorption. Repeatedly, the adsorption-desorption experiment was conducted for 10 cycles.

Competitive adsorption experiments. 30 mL of THF, 30 mL of 2-methyltetrahydrofuran (MTHF), and a mixture of 15 mL of THF with 15 mL MTHF were loaded respectively into three glass bottles. The glass tubes with 10 mg of activated powder of racemic **D6H** were placed in each bottle. The glass bottles were heated at 75 °C for 2 days.

30 mL of DIPE, 30 mL of *n*-hexane (NH), and a mixture of 15 mL of DIPE with 15 mL NH were loaded respectively into three glass bottles. The glass tubes with 10 mg of activated powder of racemic **D6H** were placed in each bottle. The glass bottles were heated at 70 °C for 2 days.

30 mL of DOX, 30 mL of CYH, and a mixture of 15 mL of DOX with 15 mL CYH were loaded respectively into three glass bottles. The glass tubes with 10 mg of activated powder of racemic **D6H** were placed in each bottle. The glass bottles were heated at 90 °C for 2 days. All the absorption capacity was finally estimated by NMR.

Calculation of pore volume (V_p). The V_p was calculated with the equation:

$$V_P = \frac{xM}{V_m \rho} \cdot 10^{-3}$$

where x is the maximum uptake of THF, M is the relative molecular mass, V_m is the molar volume of gas in the standard state, ρ is the density of liquid THF in the case that the THF molecules are capillary-condensed in the pores.

According to the adsorption isotherms of THF (Fig. 5a), x is 125.43 cm^3_{STP}/g taken at p/p_0 = 0.914. Consequently, V_p is calculated to be 0.453 cm^3/g.

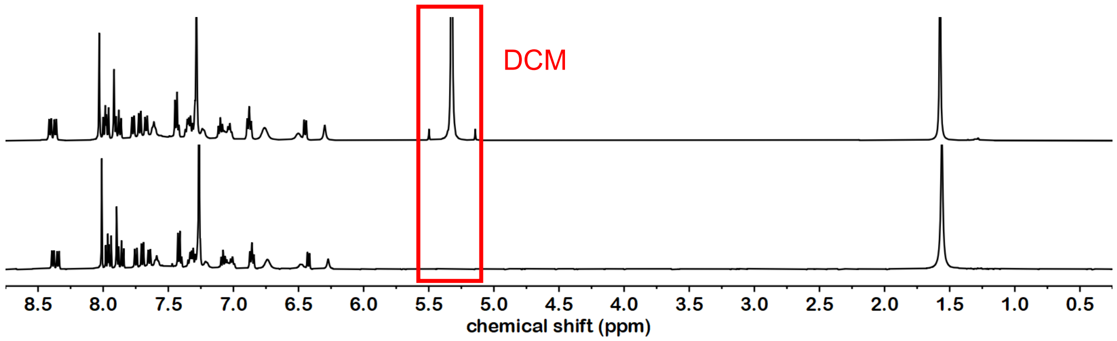

Supplementary Fig. 16 | ^{1}H NMR spectrum of powder of racemic **D6H** dried from DCM before and after activation (500 MHz, $CDCl_3$, 298 K).

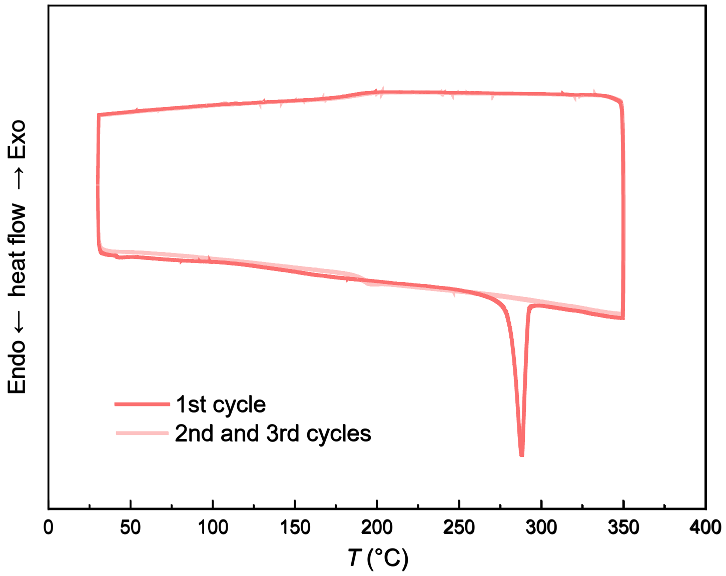

Supplementary Fig. 17 | DSC curves of the activated powder of racemic **D6H**.

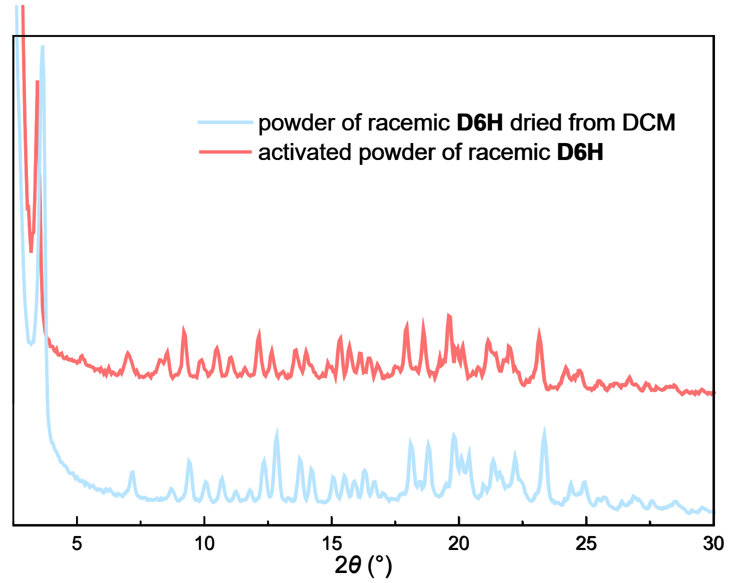

Supplementary Fig. 18 | PXRD patterns of powder of racemic **D6H** powder dried from DCM before and after activation.

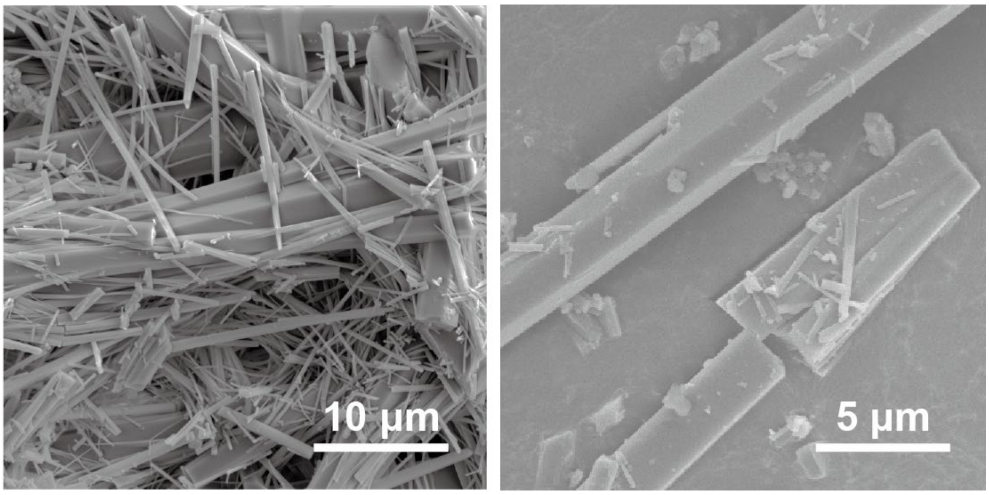

Supplementary Fig. 19 | SEM images of the activated powder of racemic **D6H**.

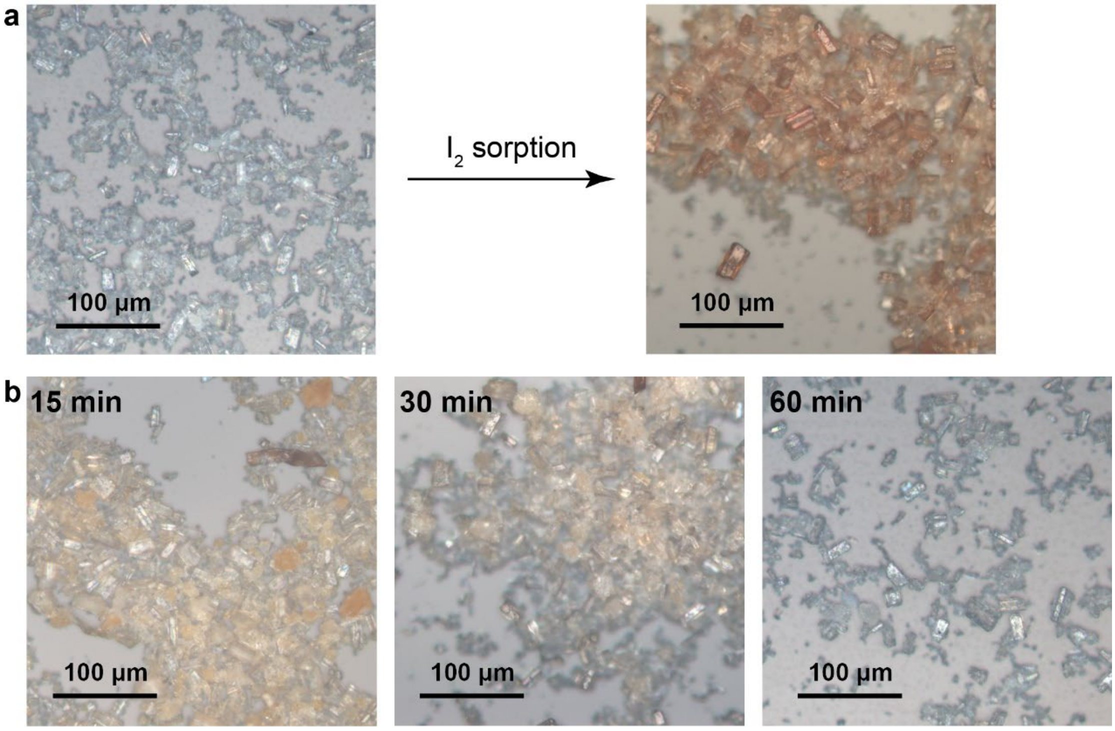

Supplementary Fig. 20 | Adsorption and desorption of iodine by the activated powder of racemic **D6H**. **a**, Optical microscope images of the activated powder of racemic **D6H** before and after being soaked in an ethanol solution of iodine (0.1 mol/L). **b**, Optical microscope images showing the desorption of iodine from the activated powder of racemic **D6H**.

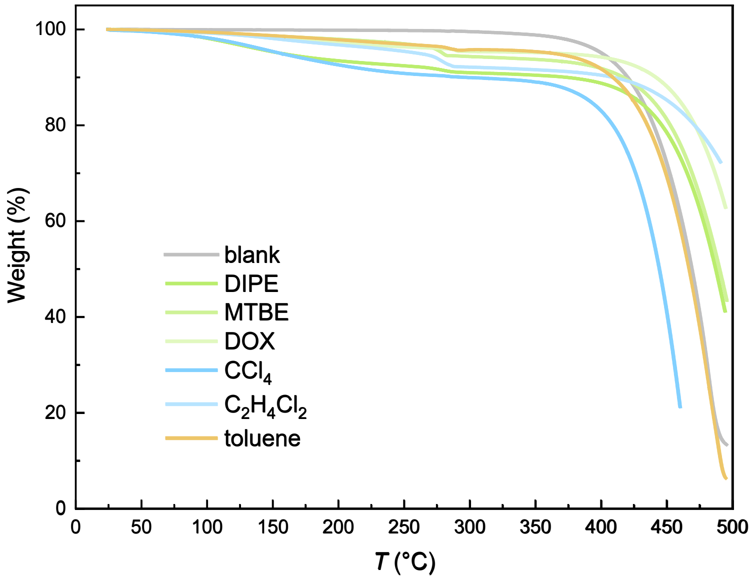

Supplementary Fig. 21 | TGA curves of the activated powder of racemic **D6H** before and after the adsorption of DIPE, MTBE, DOX, CCl_4, $C_2H_4Cl_2$ and toluene.

Supplementary Table 4 | Adsorption capacity of the activated powder of racemic **D6H** toward various solvents (analyzed by TGA).

Solvent	Weight loss of solvent*	Mole ratio (**D6H** : solvent)	Adsorption capacity (mmol/g)
THF	7.18%	1 : 0.76	1.075
DIPE	9.30%	1 : 0.71	1.004
MTBE	6.02%	1 : 0.49	0.693
DOX	4.68%	1 : 0.40	0.566
$CHCl_3$	10.87%	1 : 0.70	0.990
CCl_4	10.52%	1 : 0.53	0.750
$C_2H_4Cl_2$	7.51%	1 : 0.58	0.821
toluene	4.58%	1 : 0.37	0.523

*The weight loss was calculated according to the value at 285 °C.

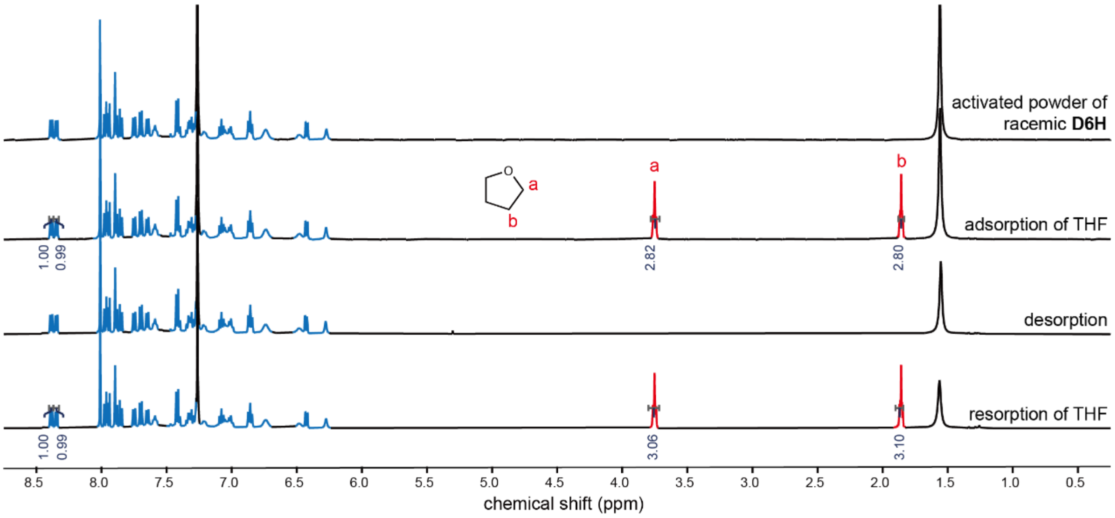

Supplementary Fig. 22 | ^1H NMR spectrum of the activated powder of racemic **D6H** upon adsorption and desorption of THF (500 MHz, $CDCl_3$, 298 K).

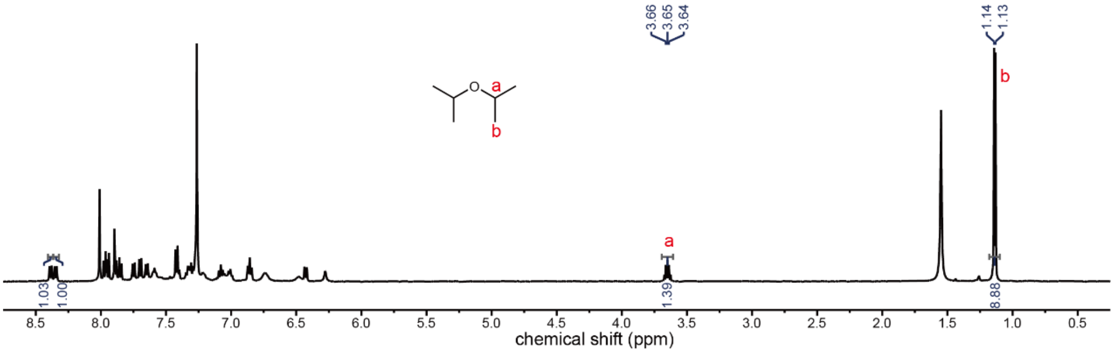

Supplementary Fig. 23 | ^{1}H NMR spectrum of the activated powder of racemic **D6H** after adsorption of DIPE (500 MHz, $CDCl_3$, 298 K).

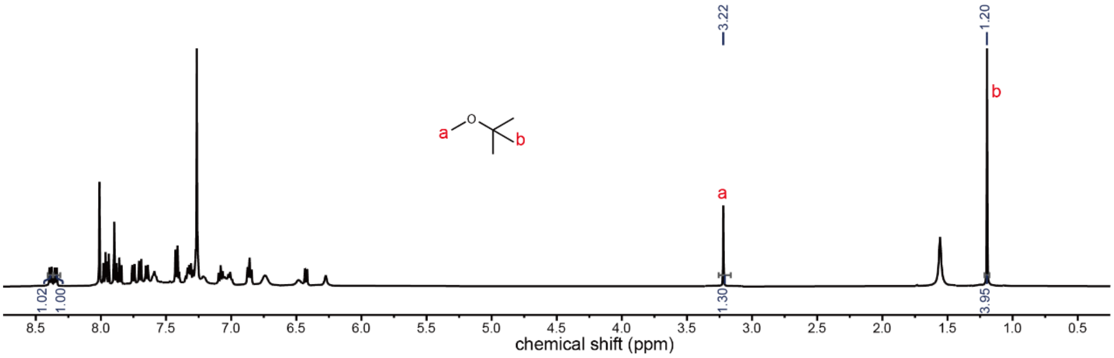

Supplementary Fig. 24 | ^{1}H NMR spectrum of the activated powder of racemic **D6H** after adsorption of MTBE (500 MHz, $CDCl_3$, 298 K).

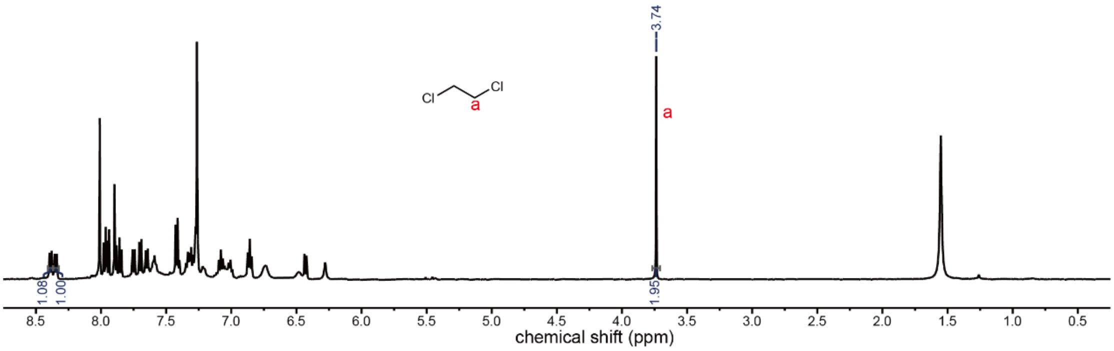

Supplementary Fig. 25 | ^{1}H NMR spectrum of the activated powder of racemic **D6H** after adsorption of DOX (500 MHz, $CDCl_3$, 298 K).

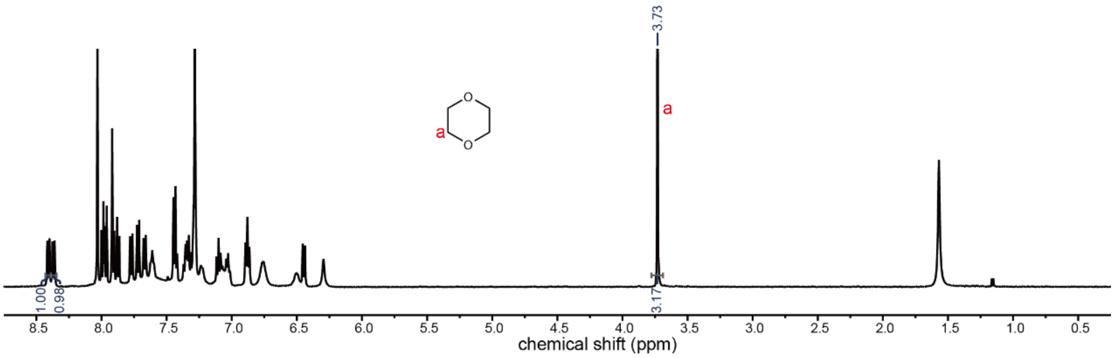

Supplementary Fig. 26 | ^{1}H NMR spectrum of the activated powder of racemic **D6H** after adsorption of $C_2H_4Cl_2$ (500 MHz, $CDCl_3$, 298 K).

Supplementary Table 5 | Adsorption capacity of the activated powder of racemic **D6H** toward various solvents (analyzed by NMR).

Solvent	Mole ratio (**D6H** : THF)	Adsorption capacity (mmol/g)
THF	1 : 0.71	1.004
DIPE	1 : 0.70	0.990
MTBE	1 : 0.44	0.622
DOX	1 : 0.39	0.552
$C_2H_4Cl_2$	1 : 0.49	0.693

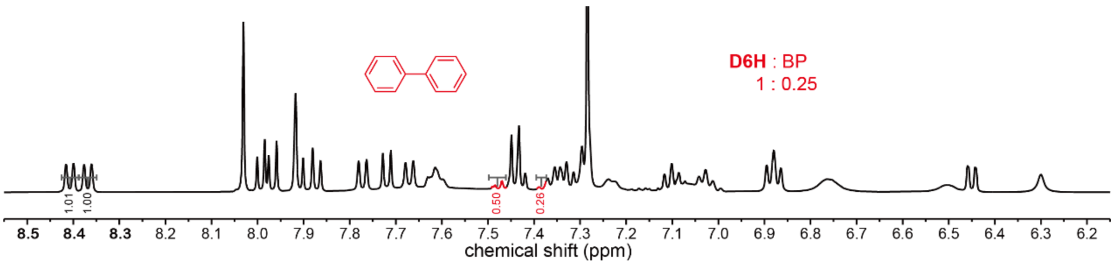

Supplementary Fig. 27 | ^{1}H NMR spectrum of the activated powder of racemic **D6H** after adsorption of BP (500 MHz, $CDCl_3$, 298 K).

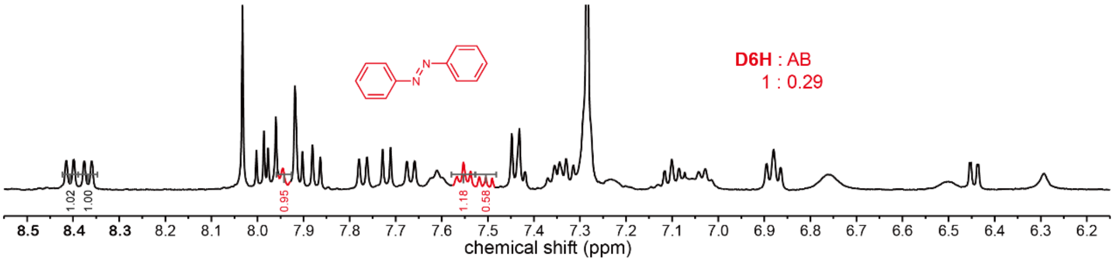

Supplementary Fig. 28 | ^{1}H NMR spectrum of the activated powder of racemic **D6H** after adsorption of AB (500 MHz, $CDCl_3$, 298 K).

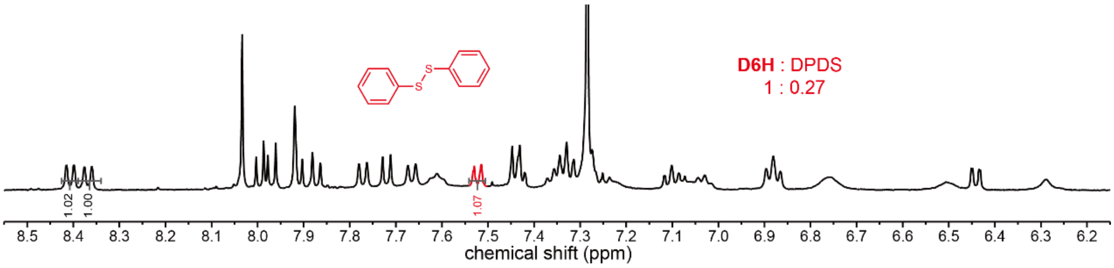

Supplementary Fig. 29 | ^{1}H NMR spectrum of the activated powder of racemic **D6H** after adsorption of DPDS (500 MHz, $CDCl_3$, 298 K).

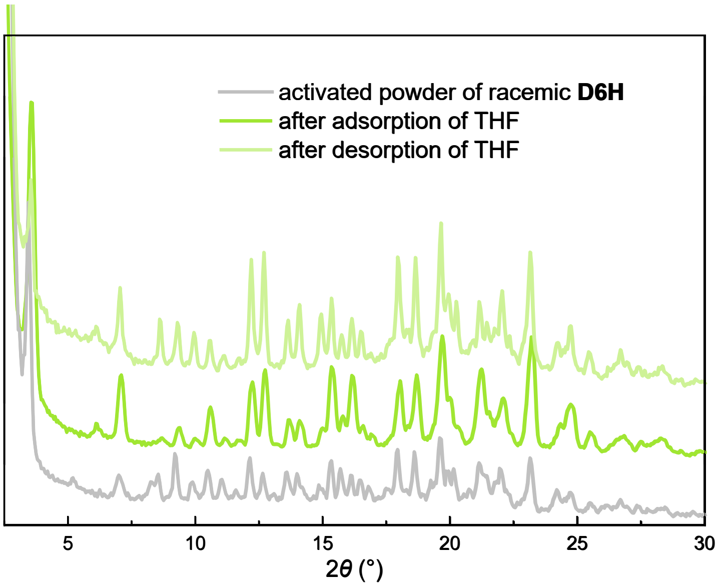

Supplementary Fig. 30 | PXRD patterns of the activated powder of racemic **D6H** upon adsorption and desorption of THF.

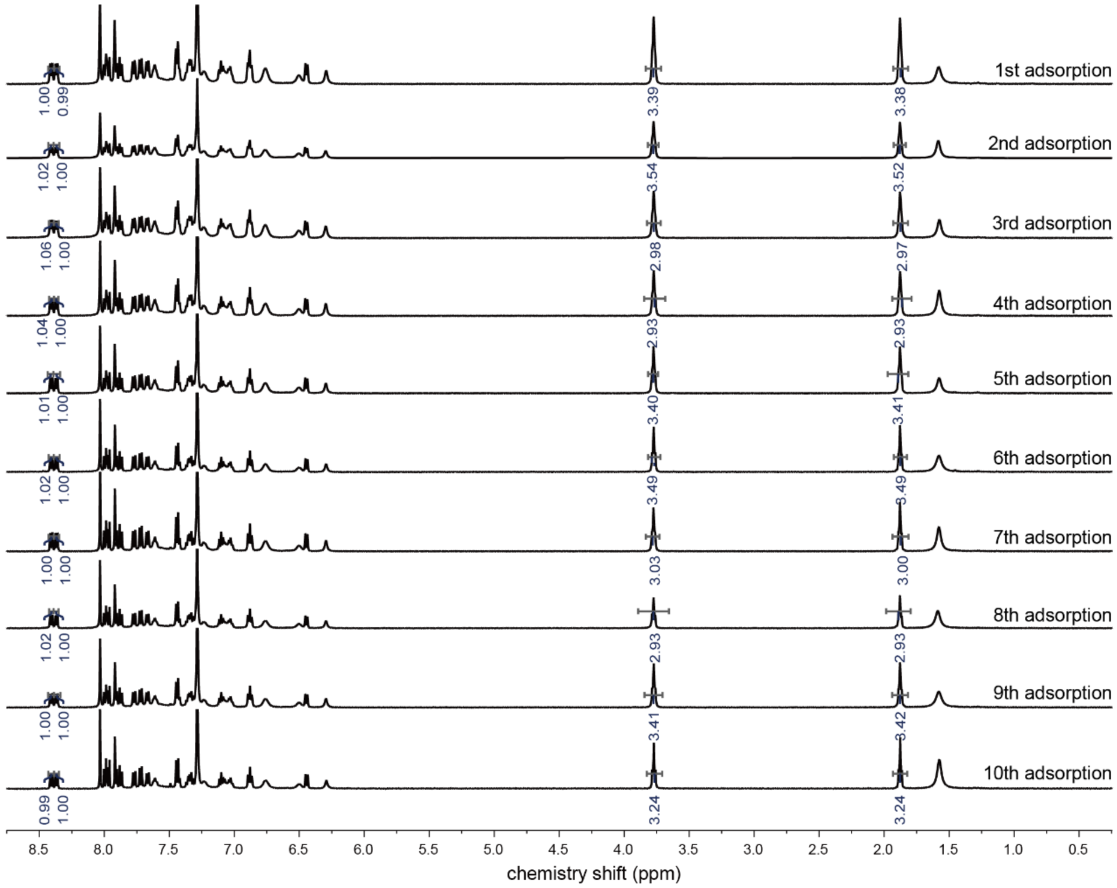

Supplementary Fig. 31 | ^{1}H NMR spectra showing the adsorption of THF by the activated powder of racemic **D6H** after various adsorption-desorption cycles (500 MHz, $CDCl_3$, 298 K).

Supplementary Table 6 | Adsorption capacity of the activated powder of racemic **D6H** after various adsorption-desorption cycles (analyzed by NMR).

Number of cycles	Mole ratio (**D6H** : THF)	Adsorption capacity (mmol/g)
1	1 : 0.85	1.202
2	1 : 0.88	1.245
3	1 : 0.75	1.061
4	1 : 0.73	1.033
5	1 : 0.85	1.202
6	1 : 0.87	1.231
7	1 : 0.76	1.075
8	1 : 0.73	1.032
9	1 : 0.85	1.202
10	1 : 0.81	1.146

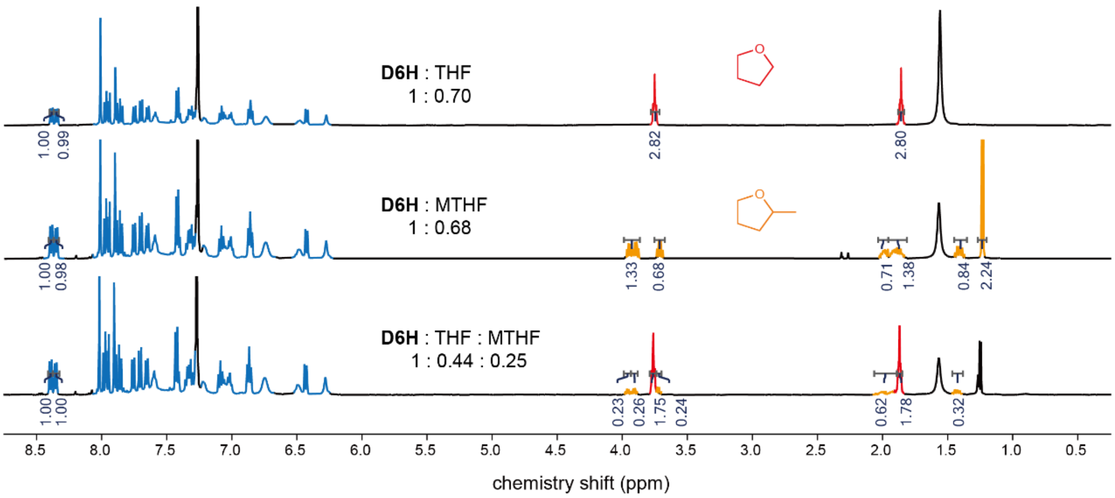

Supplementary Fig. 32 | ^{1}H NMR spectra of the activated powder of racemic **D6H** after adsorption of THF, MTHF and a blend vapor of THF with MTHF.

Supplementary Table 7 | Adsorption capacity of the activated powder of racemic **D6H** toward THF, MTHF and a blend vapor of THF with MTHF (analyzed by NMR).

Solvent		Mole ratio (**D6H** : THF)	Adsorption capacity (mmol/g)
THF		1 : 0.70	0.990
MTHF		1 : 0.68	0.961
Blend vapor	THF	1 : 0.44	0.622
	MTHF	1 : 0.25	0.354

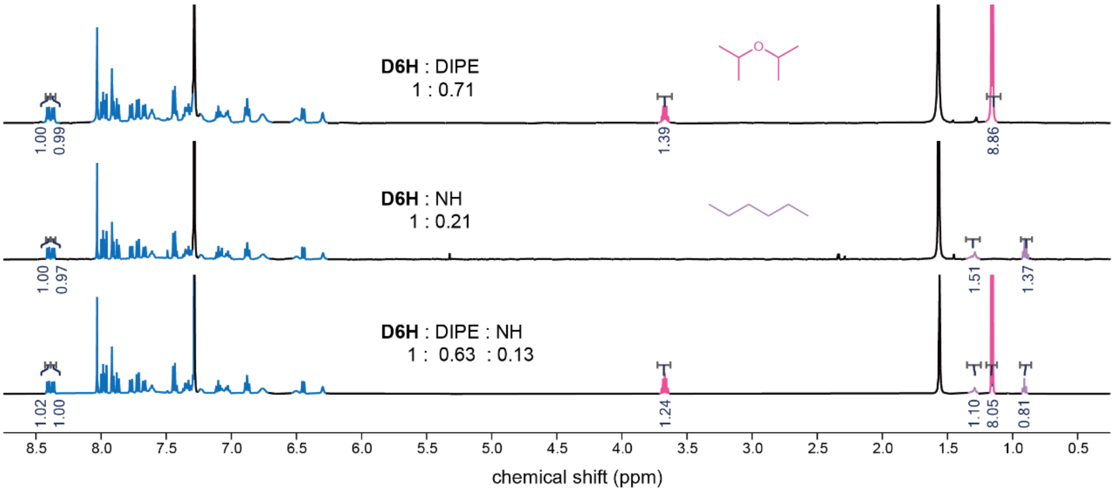

Supplementary Fig. 33 | ^{1}H NMR spectra of the activated powder of racemic **D6H** after adsorption of DIPE, NH and a blend vapor of DIPE with NH.

Supplementary Table 8 | Adsorption capacity of the activated powder of racemic **D6H** toward DIPE, NH and a blend vapor of DIPE with NH (analyzed by NMR).

Solvent		Mole ratio (**D6H** : THF)	Adsorption capacity (mmol/g)
DIPE		1 : 0.71	1.004
NH		1 : 0.21	0.297
Blend vapor	DIPE	1 : 0.63	0.891
	NH	1 : 0.13	0.184

Supplementary Table 9 | Adsorption capacity of the activated powder of racemic **D6H** toward DOX, CYH, and a blend vapor of DOX and CYH (analyzed by NMR).

Solvent		Mole ratio (**D6H** : THF)	Adsorption capacity (mmol/g)
DOX		1 : 0.39	0.552
CYH		1 : 0.05	0.071
Blend vapor	DOX	1 : 0.29	0.410
	CYH	1 : 0.01	0.014

Supplementary Table 10 | Crystallographic data and structure refinement for THF@**D6H** (CCDC 2259036).

Empirical formula	$C_{60}H_{42}O$
Formula weight	779.001
Temperature (K)	173.0
Crystal system	Trigonal
Space group	*R*-3
a (Å)	49.9555(10)
b (Å)	49.9555(10)
c (Å)	10.5017(3)
α (°)	90
β (°)	90
γ (°)	120
Volume (Å^3)	22696.4(9)
Z	18
$\rho_{calc.}$ (g·cm^{-3})	1.026
μ (mm^{-1})	0.454
F(000)	7401.7
Crystal size (mm^3)	0.5 × 0.1 × 0.1
Radiation	Cu Kα (λ = 1.54178)
2*Θ* range for data collection (°)	7.08 to 130
Index ranges	$-55 \leq h \leq 58$, $-58 \leq k \leq 58$, $-12 \leq l \leq 12$
Reflections collected	33801
Independent reflection	7502 [R_{int} = 0.1080, R_σ = 0.0812]
Data/restraints/parameters	7502/0/550
Goodness-of-fit on F^2	1.004
Final *R* indexes [$I \geq 2\sigma(I)$]	R_1 = 0.0683, wR_2 = 0.1927
Final *R* indexes [all data]	R_1 = 0.0926, wR_2 = 0.2118
Largest diff. peak/hole (e·Å^{-3})	0.57/−0.40

Note: In each asymmetric unit, only one THF molecules could be found and identified, and the other possible existing THF and *n*-pentane molecules in a disordered fashion was removed by solvent mask using Olex2.

- Explanation for the Alert A and Alert B

  PLAT029_ALERT_3_A Diffrn measured fraction theta full value low 0.874

  PLAT911_ALERT_3_B Missing FCF Refl Between Thmin & STh/L = 0.588 1092

—— The Alert_3_A and Alert_3_B were majorly caused by the low quality of the single crystal and the volatilization of THF molecules confined in the pores. However, this would not affect the structural determination in this study.

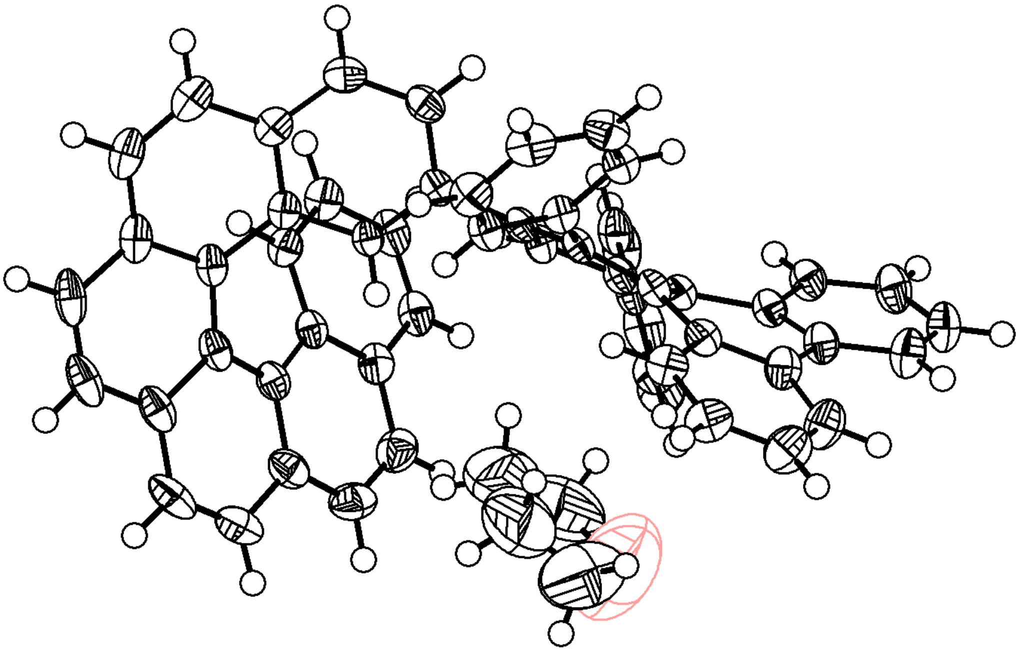

Supplementary Fig. 34 | X-ray crystallographic structure of THF@**D6H** (CCDC 2259036) (only *M* enantiomer is shown). Thermal ellipsoids are shown at a 50% probability level. The identification for the oxygen atom of THF is assisted by theoretical calculations (See theoretical calculation part).

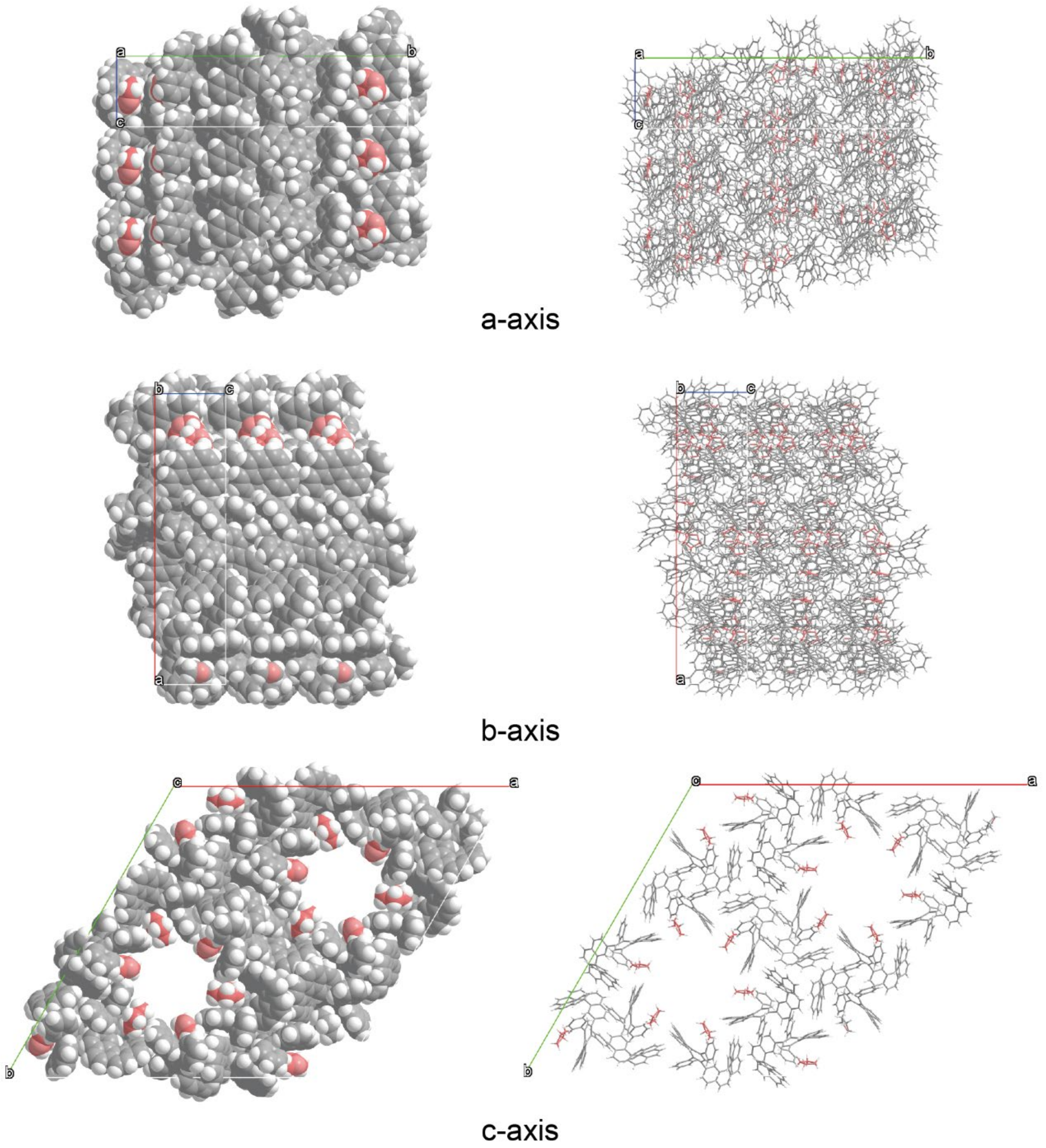

Supplementary Fig. 35 | Molecular packing of THF and **D6H** in the crystal. The C atoms of THF are marked in red.

Supplementary Table 11 | Crystallographic data and structure refinement for $CHCl_3$@**D6H** (CCDC 2255046).

Empirical formula	$C_{57}H_{35}Cl_3$
Formula weight	826.20
Temperature (K)	233.0
Crystal system	Trigonal
Space group	*R*-3
a (Å)	50.275(3)
b (Å)	50.275(3)
c (Å)	10.5646(6)
α (°)	90
β (°)	90
γ (°)	120
Volume (Å^3)	23125(3)
Z	18
$\rho_{calc.}$ (g·cm^{-3})	1.068
μ (mm^{-1})	1.858
F(000)	7704.0
Crystal size (mm^3)	0.1 × 0.02 × 0.02
Radiation	Cu Kα (λ = 1.54178)
2*Θ* range for data collection (°)	3.514 to 135.82
Index ranges	$-60 \leq h \leq 59$, $-60 \leq k \leq 56$, $-11 \leq l \leq 12$
Reflections collected	95432
Independent reflection	9342 [R_{int} = 0.1362, R_σ = 0.0619]
Data/restraints/parameters	9342/42/542
Goodness-of-fit on F^2	0.984
Final *R* indexes [$I \geq 2\sigma(I)$]	R_1 = 0.0823, wR_2 = 0.2292
Final *R* indexes [all data]	R_1 = 0.1304, wR_2 = 0.2767
Largest diff. peak/hole (e·Å^{-3})	0.40/−0.50

Note: In each asymmetric unit, only one $CHCl_3$ molecules could be found and identified, and the other possible existing $CHCl_3$ and *n*-pentane molecules in a disordered fashion was removed by solvent mask using Olex2.

- Explanation for the Alert B

  PLAT260_ALERT_2_B Large Average Ueq of Residue Including Cl1 0.695

—— The Alert_2_B was majorly caused by the low quality of the single crystal and the volatilization of $CHCl_3$ molecules confined in the pores. However, this would not affect the structural determination in this study.

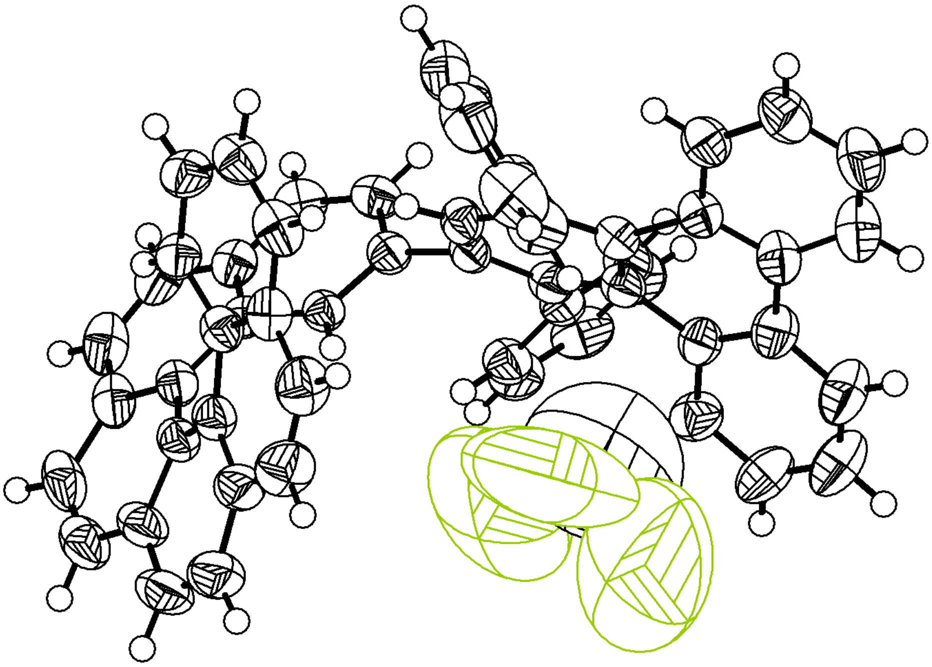

Supplementary Fig. 36 | X-ray crystallographic structure of $CHCl_3$@**D6H** (CCDC 2255046) (only *P* enantiomer is shown). Thermal ellipsoids are shown at a 50% probability level.

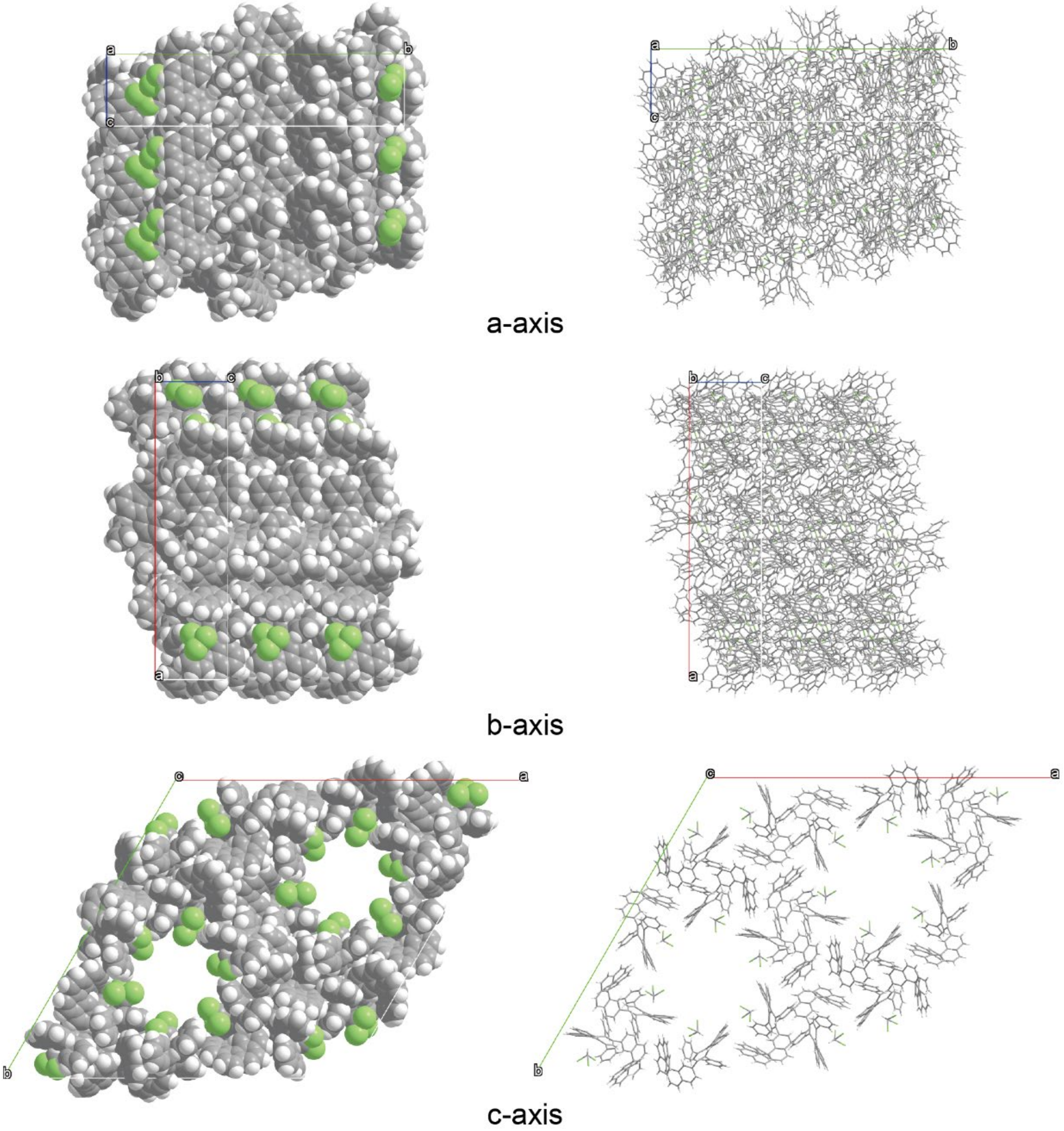

Supplementary Fig. 37 | Molecular packing of $CHCl_3$ and **D6H** in the crystal.

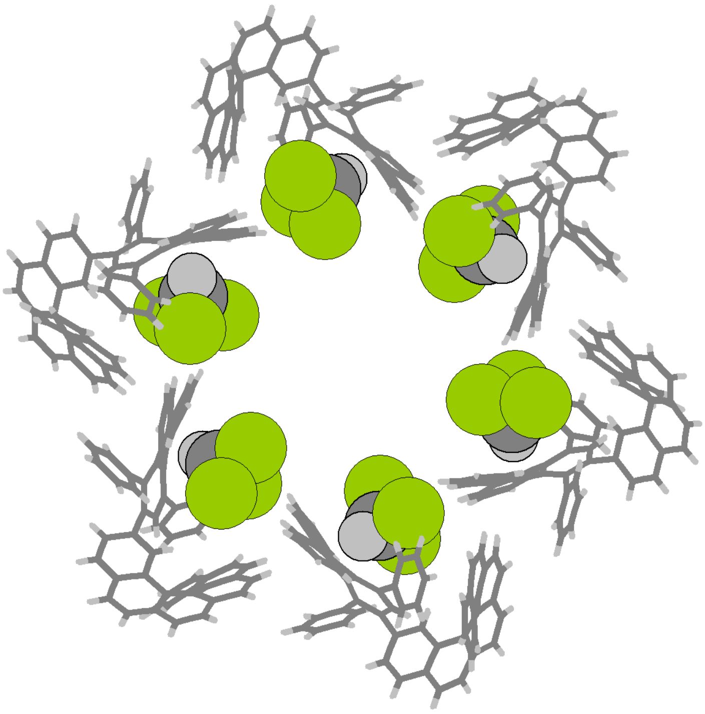

Supplementary Fig. 38 | Cross-section of a channel containing six $CHCl_3$ molecules.

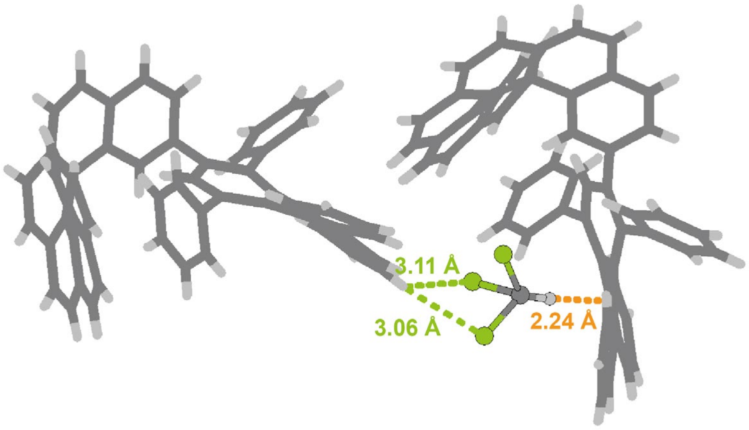

Supplementary Fig. 39 | Intermolecular interactions between the adsorbed $CHCl_3$ and **D6H** molecules.

4. Theoretical calculations

Density functional theory (DFT) calculations were carried out using CP2K 8.2 program[2]. For **D6H** framework without any adsorbed small molecules, the geometry optimization was performed on both structure and cell at the PBE0-D3(BJ)[3,4]/DZVP-MOLOPT-SR-GTH[5] level of theory using primitive cell of the X-ray single crystal structure as the first guess. For the geometry optimizations of **D6H** frameworks with adsorbed THF or $CHCl_3$ molecules, the cell parameters for the first guesses were set as same as the optimized **D6H** structure, and were fixed at the subsequent structure optimizations.

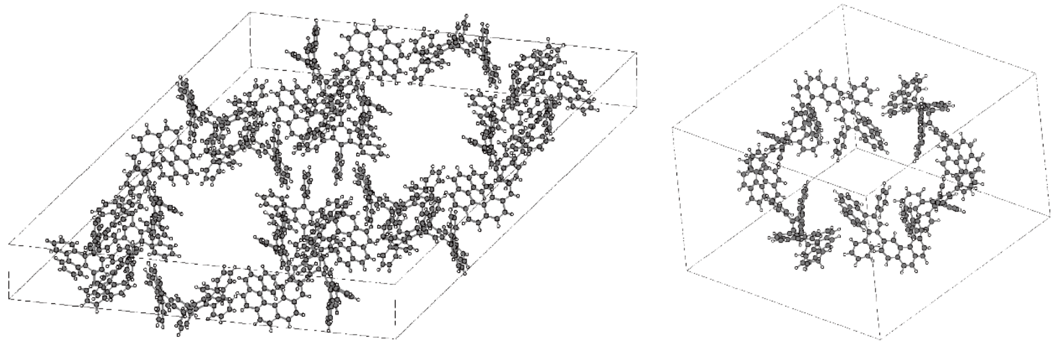

Supplementary Fig. 40 | A crystal cell of **D6H** containing 18 molecules and a primitive cell for theoretical calculation containing 6 molecules.

For the calculation of noncovalent interaction (NCI) analysis, we firstly selected the relevant molecules from the optimized framework structures (for example, two neighboring **D6H** molecules), and then calculated the single point for on the structures without geometry optimization to give the electronic structure information. The single point calculations were carried out using Gaussian 16 program[6] at the PBE0-D3(BJ)[3,4]/def2-TZVPP[7] level of theory. Subsequently, the calculations of noncovalent interaction (NCI) analysis[8] were performed using Multiwfn 3.8-dev program[9,10]. Cutoff of the electron density ρ was set to 0.05. The NCI map with the isosurface of the reduced density gradient (RDG) mapped by the electron density multiplied by the sign of the second Hessian eigenvalue function (sign(λ_2)ρ) were visualized by using VMD program[11]. Isosurface value was set to 0.6 with a color scale of $-0.04 < \text{sign}(\lambda_2)\rho < 0.02$.

When solving the single-crystal structures of THF@**D6H**, we found a regular five-membered ring formed by five Q peaks which could be assigned to a THF molecule (Supplementary Fig. 41, Q peaks are marked in yellow). However, we could not distinguish which Q peak should be the oxygen atom. Therefore, we made theoretical calculations by building five initial guesses with oxygen on each Q peak respectively (namely, THF@**D6H**_1, THF@**D6H**_2, THF@**D6H**_3, THF@**D6H**_4 and THF@**D6H**_5) (Supplementary Fig. 42). The initial guesses were optimized at the PBE0-D3(BJ) /DZVP-MOLOPT-SR-GTH level. The result indicated that the configuration THF@**D6H**_1 gave the lowest electron energy (Supplementary Table 12), thus appeared to be the most plausible configuration for the single crystal structure.

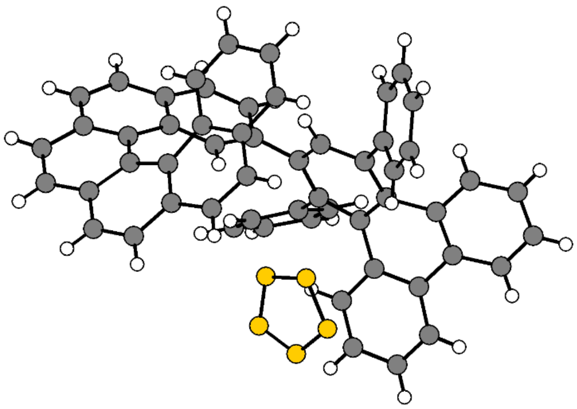

Supplementary Fig. 41 | Intermediate structure of the single crystal refinement for THF@**D6H**. In the smallest symmetry unit, there are a **D6H** molecule and a THF molecule appearing as a pentagon without hydrogen atoms (marked in yellow).

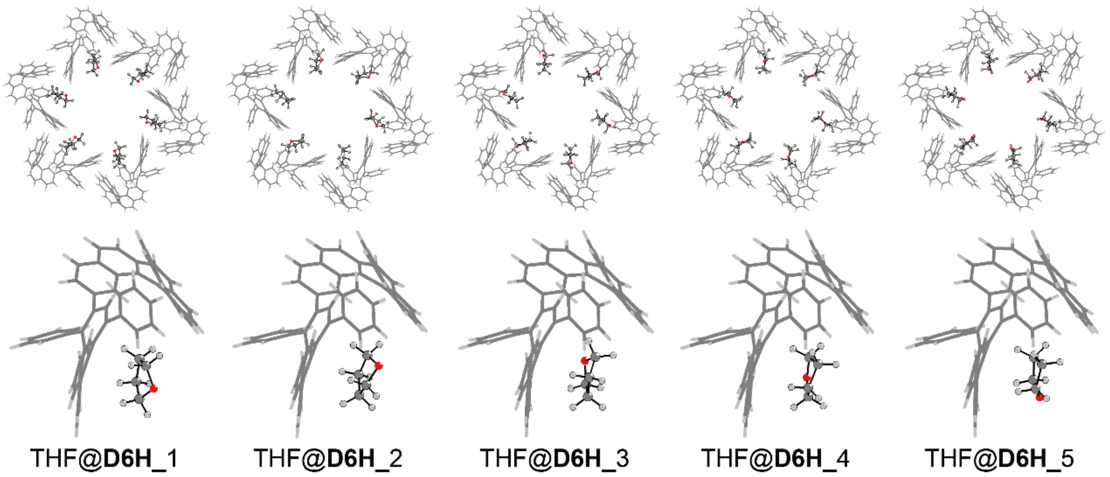

Supplementary Fig. 42 | Five possible configurations of THF@**D6H** depending on the position of the oxygen atoms

Supplementary Table 12 | Calculation results for the electron energies of configurations of THF@**D6H**.

Configuration	Electron energy* (Hartree)	Electron energy** (kcal/mol)
THF@**D6H**_1	-2294.02952	0
THF@**D6H**_2	-2294.02708	1.5311
THF@**D6H**_3	-2293.99997	18.5426
THF@**D6H**_4	-2294.02798	0.9663
THF@**D6H**_5	-2294.02686	1.6691

*The calculations were performed at the PBE0-D3(BJ)/def2-TZVPP//PBE0-D3(BJ)/DZVP-MOLOPT-SR-GTH level of theory.

The electron energy of THF@D6H**_1 is set as 0 kcal/mol.

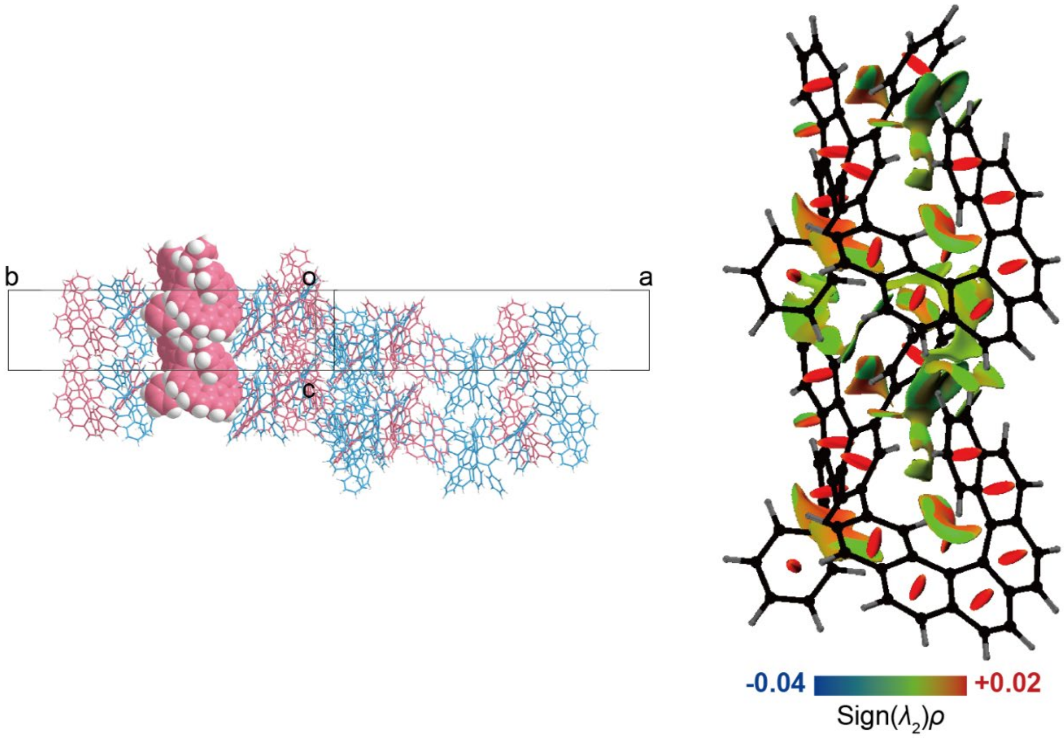

Supplementary Fig. 43 | NCI maps for the intermolecular interactions between homochiral **D6H** molecules arranged in parallel along the c-axis, and their relative positions marked in space-fill representation (left).

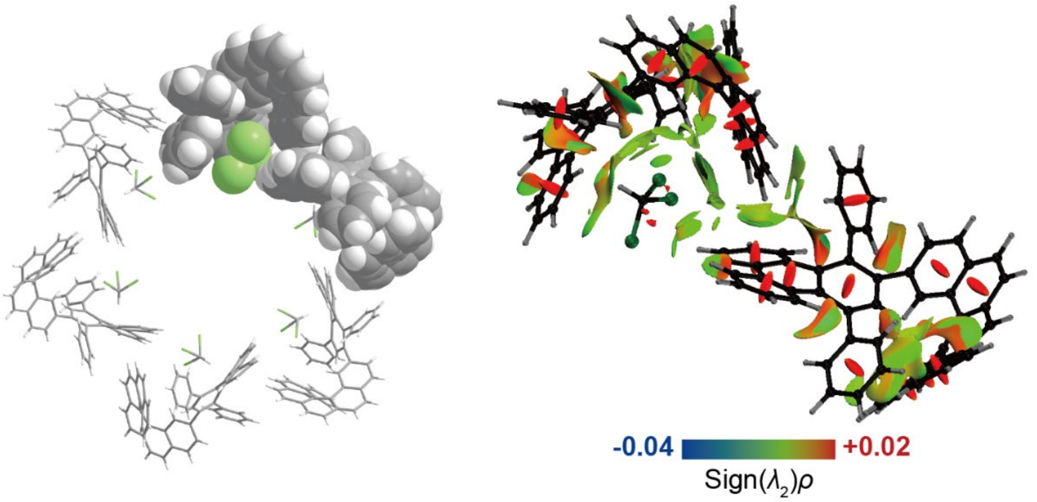

Supplementary Fig. 44 | NCI maps for the intermolecular interactions between $CHCl_3$ and two **D6H** molecules nearby and their relative positions marked in space-fill representation (left).

Supplementary References

1 Shen, C. et al. Oxidative cyclo-rearrangement of helicenes into chiral nanographenes. *Nat. Commun.* **12**, 2786–2793 (2021).

2 Kuehne, T. D. et al. CP2K: An electronic structure and molecular dynamics software package - Quickstep: Efficient and accurate electronic structure calculations *J. Chem. Phys.* **152**, 194103 (2020).

3 Adamo, C. & Barone, V. Toward reliable density functional methods without adjustable parameters: The PBE0 model. *J. Chem. Phys.* **110**, 6158–6170 (1999).

4 Grimme, S., Antony, J., Ehrlich, S. & Krieg, H. A consistent and accurate *ab initio* parametrization of density functional dispersion correction (DFT-D) for the 94 elements H-Pu. *J. Chem. Phys.* **132**, 154104 (2010).

5 VandeVondele, J. & Hutter, J. Gaussian basis sets for accurate calculations on molecular systems in gas and condensed phases. *J. Chem. Phys.* **127**, 114105 (2007).

6 Frisch, M. J. et al. Gaussian, Inc., Gaussian 16, Revision A.03. *Wallingford CT* (2016).

7 Weigend, F. & Ahlrichs, R. Balanced basis sets of split valence, triple zeta valence and quadruple zeta valence quality for H to Rn: Design and assessment of accuracy. *Phys. Chem. Chem. Phys.* **7**, 3297–3305 (2005).

8 Johnson, E. R. et al. Revealing noncovalent interactions. *J. Am. Chem. Soc.* **132**, 6498–6506 (2010).

9 Lu, T. & Chen, F. Multiwfn: A multifunctional wavefunction analyzer. *J. Comput. Chem.* **33**, 580–592 (2012).

10 Lu, T. & Chen, Q. Van der waals potential: An important complement to molecular electrostatic potential in studying intermolecular interactions. *J. Mol. Model.* **26**, 315 (2020).

11 Humphrey, W., Dalke, A. & Schulten, K. VMD - Visual molecular dynamics. *J. Molec. Graphics* **14**, 33–38 (1996).
